# Exosome-dependent immune surveillance at the metastatic niche requires BAG6 and CBP/p300-dependent acetylation of p53

**DOI:** 10.7150/thno.36378

**Published:** 2019-08-14

**Authors:** Maximiliane Schuldner, Bastian Dörsam, Olga Shatnyeva, Katrin S. Reiners, Andriy Kubarenko, Hinrich P. Hansen, Florian Finkernagel, Katrin Roth, Sebastian Theurich, Andrea Nist, Thorsten Stiewe, Annette Paschen, Gero Knittel, Hans C. Reinhardt, Rolf Müller, Michael Hallek, Elke Pogge von Strandmann

**Affiliations:** 1 Experimental Tumor Research, Center for Tumor Biology and Immunology, Clinic for Hematology, Oncology and Immunology, Philipps University, Marburg, Germany; 2 Innate Immunity Group, Department I of Internal Medicine, University Hospital of Cologne, Cologne, Germany; 3 Institute of Clinical Chemistry and Clinical Pharmacology, University Hospital Bonn, Bonn, Germany; 4 Institute of Molecular Biology and Tumor Research (IMT), Center for Tumor Biology and Immunology (ZTI), Philipps University, Marburg, Germany; 5 Center for Tumor Biology and Immunology (ZTI), Core Facility Cellular Imaging, Philipps University, Marburg, Germany; 6 Cancer and Immunometabolism Research Group, Department I of Internal Medicine, University Hospital of Cologne, Germany (current address: Department of Medicine III, University Hospital Munich, LMU, Germany); 7 Institute of Molecular Oncology and Genomics Core Facility, Center for Tumor Biology and Immunology (ZTI), Philipps University Marburg, Germany; 8 Department of Dermatology, University Hospital Essen, Essen and German Cancer Consortium (DKTK), Partner site University Hospital Essen, Essen, Germany; 9 Clinic I of Internal Medicine, University Hospital of Cologne, Cologne, Germany.; 10 Cologne Excellence Cluster on Cellular Stress Response in Aging-Associated Diseases (CECAD), University of Cologne, Cologne, Germany.; 11 Center of Integrated Oncology (CIO), University Hospital of Cologne, Cologne, Germany.; 12 Center for Molecular Medicine Cologne (CMMC), University of Cologne, Cologne, Germany

**Keywords:** extracellular vesicles, exosomes, BAG6/CBP/p300-p53, metastasis, melanoma

## Abstract

Extracellular vesicles released by tumor cells contribute to the reprogramming of the tumor microenvironment and interfere with hallmarks of cancer including metastasis. Notably, melanoma cell-derived EVs are able to establish a pre-metastatic niche in distant organs, or on the contrary, exert anti-tumor activity. However, molecular insights into how vesicles are selectively packaged with cargo defining their specific functions remain elusive.

**Methods**: Here, we investigated the role of the chaperone Bcl2-associated anthogene 6 (BAG6, synonym Bat3) for the formation of pro- and anti-tumor EVs. EVs collected from wildtype cells and BAG6-deficient cells were characterized by mass spectrometry and RNAseq. Their tumorigenic potential was analyzed using the B-16V transplantation mouse melanoma model.

**Results**: We demonstrate that EVs from B-16V cells inhibit lung metastasis associated with the mobilization of Ly6C^low^ patrolling monocytes. The formation of these anti-tumor-EVs was dependent on acetylation of p53 by the BAG6/CBP/p300-acetylase complex, followed by recruitment of components of the endosomal sorting complexes required for transport (ESCRT) via a P(S/T)AP double motif of BAG6. Genetic ablation of BAG6 and disruption of this pathway led to the release of a distinct EV subtype, which failed to suppress metastasis but recruited tumor-promoting neutrophils to the pre-metastatic niche.

**Conclusion**: We conclude that the BAG6/CBP/p300-p53 axis is a key pathway directing EV cargo loading and thus a potential novel microenvironmental therapeutic target.

## Introduction

Extracellular vesicles (EVs) are known to stimulate tumor progression and to interfere with hallmarks of cancer including immune evasion and metastasis 1. Importantly, the recruitment of innate immune cells to distant organs emerged as a key process in the EV-dependent promotion of metastasis 2. A critical step in EV-mediated liver metastasis of pancreatic ductal adenocarcinomas is the MIF-dependent attraction of bone marrow-derived macrophages 3. Another example is the TLR3-mediated activation of lung epithelial cells through exosomal RNAs causing chemokine release and neutrophil recruitment 4. On the contrary, several studies propose an anti-tumor activity of EVs mainly via engagement of NK cells 5-8. Given their immune activiating potential, extracellular vesicles and exosomes are promising tools for potential clinical interventions 9-11. The molecular basis for different and opposite activities of EVs either counteracting tumor progression or supporting tumor initiation, development and metastasis 12,13 is largely unknown.

Here, we investigated the role of the chaperone Bcl2-associated anthogene 6 (BAG6, synonym Bat3) for the formation of pro- and anti-tumor EVs. BAG6 was identified as a regulator of T cell 14 and NK cell activity 15 via interaction with the TIM-3 and the NKp30 receptor, respectively. Regulation of NK cell activity depends on the presentation of BAG6 on the surface of EVs released in response to cellular stress, which triggers NK cell-mediated anti-tumor activity *in vitro* and *in vivo* 5-7. However, NKp30 is a pseudogene in the murine system and not expressed as a functional receptor 16, suggesting that the anti-tumor activity of BAG6-presenting EVs in mouse models depends on NKp30-independent acitivities.

Given the function of intracellular BAG6 in many crucial biological processes such as protein targeting and degradation 17, we hypothesize that its anti-tumor activity relies on BAG6-dependent recruitment of other effector molecules to EVs.

Not much is known about the mechanisms that regulate the formation of EVs, but ESCRT (endosomal sorting complex required for transport)-dependent and ESCRT-independent pathways are described. The cytosolic ESCRT protein complexes enable endosomal membrane remodeling that results in membrane budding and vesicle formation 18.

Using an experimental metastasis melanoma model we show that BAG6 is indispensable for the formation of anti-metastatic EVs, which are characterized by the presence of TIMP3, an inhibitor of metalloproteinases involved in the re-modelling of the extracellular matrix. These EVs from BAG6-proficient cells were able to inhibit lung metastasis associated with the accumulation of patrolling monocytes, which control metastasis 19. The release of these anti-tumor EVs required the BAG6/CBP/p300-dependent acetylation of p53 followed by the recruitment of the ESCRT machinery. Loss of BAG6 triggered a switch in the release of EVs from anti- to pro-tumorigenic EVs, which contained among others the mRNA encoding the oncogene α-catulin. Of note, these EVs generated in the absence of cellular BAG induced a neutrophil gene signature in the lungs of mice, which is indicative for the establishment of a pre-metastatic niche 20. These data provide a previously unidentified pathway, which regulates the formation of EVs.

## Results

### Cellular stress induces the release of BAG6-positive EVs

**Figure 1:**
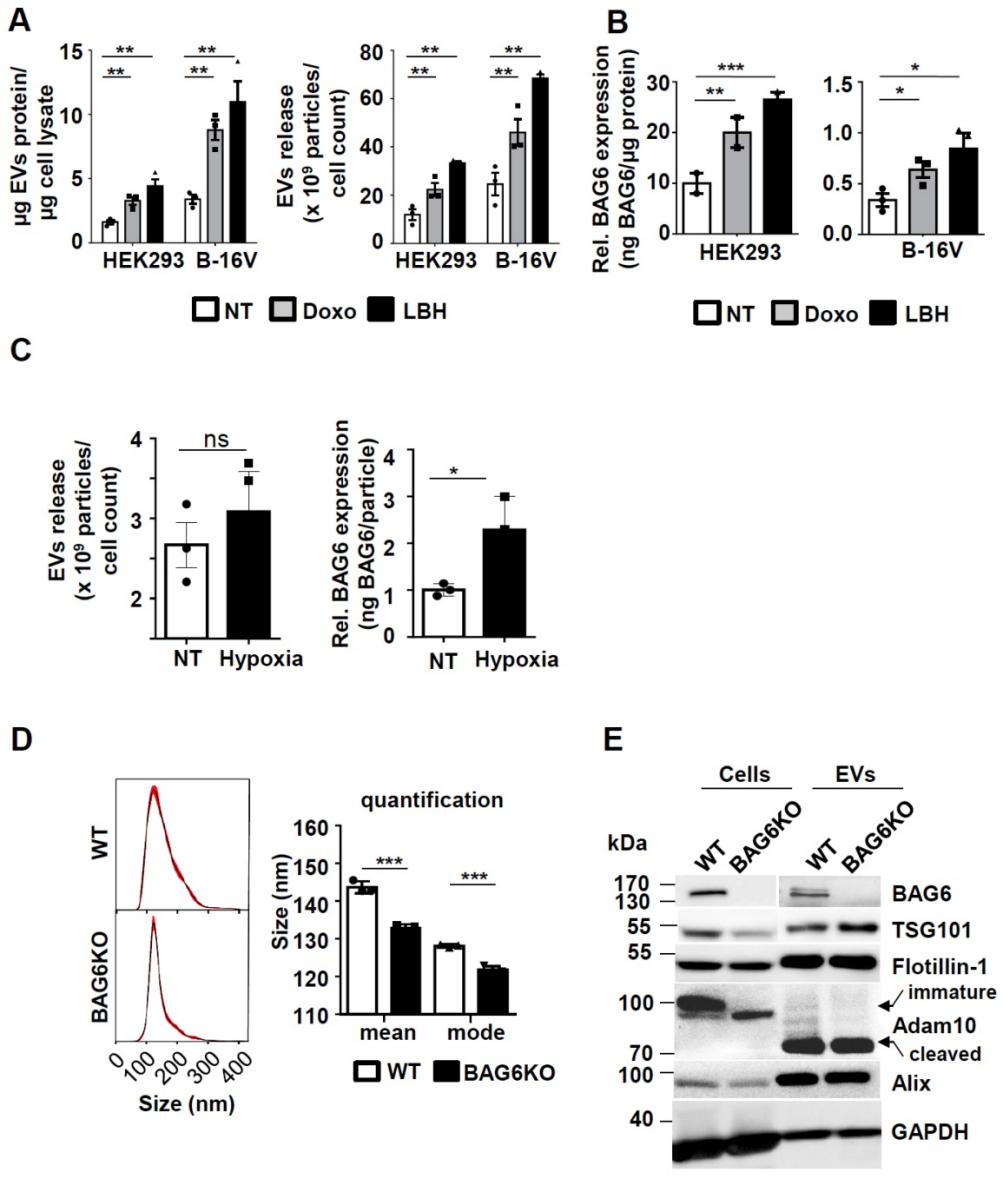
Cellular stress induces the release of BAG6-positive EVs. (A) Determination of EV protein concentration (normalized to cellular protein concentration) and particle number by NTA (normalized to cell number) of HEK293 and B-16V EVs isolated by ultracentrifugation (100k x g) of supernatants from cells either non-treated or treated with 100nM doxo or 100nM LBH for 16 hours. Bar graphs represent mean ±SEM of three independent experiments. (B) Quantification of EV-associated BAG6 by ELISA (normalized to EV protein concentration) purified by ultracentrifugation at 100k x g from HEK293 and B-16V cells that were either non-treated or treated with 100nM doxo or 100nM LBH for 16 hours. Bar graphs represent mean ±SEM of three (B-16V) and two (HEK293) independent experiments. (C) Analysis of EVs released from B-16V cells under hypoxic conditions. Left: Conditioned medium was analyzed by NTA (normalized to cell numbers) from B-16V cells incubated at either non-treated (NT) or hypoxic (1% O2) conditions. Right: Quantification of EV-associated BAG6 by ELISA (normalized to particles/ml by NTA) purified by ultracentrifugation at 100k x g from conditioned medium of B-16V cells either non-treated (NT) or cultured under hypoxic conditions (1% O2). Data represent mean ± SEM of three independent experiments (see Figure S2C for knock out data). (D) Representative particle size distribution graphs by NTA of B-16V WT- and BAG6KO-EVs and particle size quantification of the mean and mode size. The graph depicts mean ±SEM of three independent experiments. (E) Immunoblot analysis of B-16V cell lysates and EVs using the indicated antibodies. A higher exposure time was required for the detection of BAG6 in EVs. Each lane was loaded with 20 µg protein. Data are representative of two independent experiments. Two-tailed, unpaired Student's t-tests: *p < 0.05, **p < 0.01, ***p < 0.001; SEM, standard error of the mean; EVs, extracellular vesicles; kDa, kilodalton; NT, non-treated; doxo, doxorubicin; LBH, LBH-589/Panobinostat; WT, wild-type.

The enhanced release of EVs was previously shown to be associated with cellular stress, such as DNA damage 21 or with the inhibition of cellular de-acetylases 6. To analyze the underlying molecular pathways, the model cell lines HEK293 and B-16V were treated with non-lethal doses of the cytostatic drug doxorubicin (doxo) and with the histone de-acetylase inhibitor (HDACi) panabinostat (LBH) prior to purification of EVs from the cell supernatant (methods and further characterization of purified samples Figure S1). The treatment with these drugs resulted in a higher protein yield of EV preparations, which correlated with an increase of particle number in both cell lines (Figure 1A). Previous work suggested that the EV-associated ligand BAG6 is enriched on EVs released by cells upon cellular stress 5-7. In line, EVs from doxo- or LBH-treated cells were characterized by an increased amount of BAG6, irrespective of the basal expression level (Figure 1B). The inducibility of BAG6 on EVs in response to hypoxia as a melanoma-relevant stress factor has not been analyzed before. Culturing B-16V cells under 1% O_2_ for 48h increased the expression of BAG6 on EVs, while the overall secretion of EVs was not significantly enhanced (Figure 1C and S2C).

These data indicate that cellular stress induced by doxo, the HDACi LBH and hypoxia triggered the release of BAG6-enriched EVs. These EVs are referred to as stress-induced, BAG6-presenting EVs.

### mRNA and protein cargo of BAG6-proficient and -deficient EVs define different EV-subtypes

To investigate the functional role of BAG6 for EV formation, EVs were purified from wild-type (WT-EVs) and BAG6-deficient (BAG6KO-EVs) B-16V cells, which were cultured under hypoxia (1% O_2_ for 48h) (characterization of cell lines see Figure S2).

Interestingly, a slight but significant reduction of mean particle size was observed for BAG6KO-EVs compared to WT-EVs as monitored by Nanoparticle Tracking analysis (NTA) (Figure 1D), indicating that BAG6 might impact on the release of EV subpopulations. Western blot analysis of cell and EV lysates confirmed the absence of cellular and EV-associated BAG6 in BAG6KO samples, while EV markers including ESCRT components TSG101, ALIX, FLOTILLIN-1, and processed ADAM10 were detectable in both preparations (Figure 1E).

Next-generation sequencing revealed under-representation of mRNAs and over-representation of 418 mRNAs in BAG6KO-EVs compared to WT (Figure S2D). Functional gene enrichment analyses revealed that genes encoding mRNAs that were significantly decreased, or not detectable in BAG6KO-EVs, clustered predominantly in mitochondria-associated metabolic pathways (Figure S2E). In contrast, mRNAs enriched in BAG6KO-EVs were characterized by functional clusters specifically associated with immune responses, melanoma and tumour progression, the latter including vascular wall signaling, extracellular matrix remodelling and platelet activation (Figure S2E and mRNA list in Table S1).

**Figure 2:**
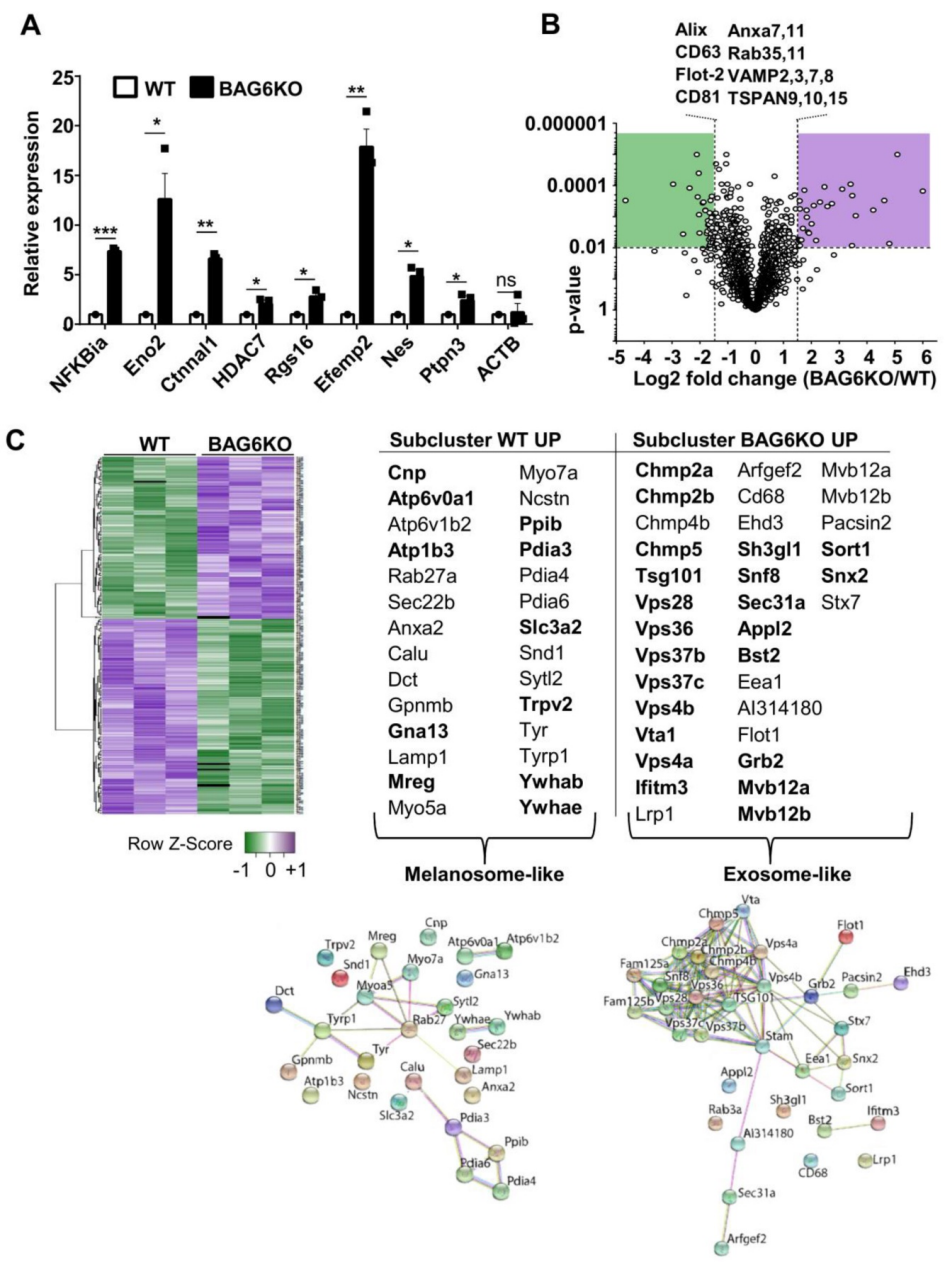
mRNA and protein cargo of BAG6-proficient and -deficient EVs define EV-subtypes. (A) qRT-PCR analysis of the indicated pro-tumorigenic markers in EVs derived from hypoxia-stressed WT or BAG6KO B-16V cells. Equal amounts of cDNA were used and the graph depicts relative expression of BAG6KO-EV Ct values normalized to the WT-EV Ct values. Bar graphs represent mean ± SEM and qRT-PCR was performed in three independent runs with three biological EV preparation replicates. (B) Volcano plot showing the log2 fold change versus p-value of the mass spectrometric analysis comparing proteins detected in EVs isolated by ultracentrifugation from the supernatant of hypoxia-stressed B-16V WT and BAG6KO cells, each measured in three independent biological replicates. The dotted lines indicate cut off lines at a p-value of 0.01 and log2 fold change of 1.5 used for analysis (significantly up- and downregulated marked in purple and green, respectively). A list of unchanged endocytic protein markers is shown. (C) Heat map showing the hierarchical clustering of WT- and BAG6KO-EV proteomic data (three independent biological replicates each, LFQ value-transformed Z scores), using Spearman Rank Correlation and Average Linkage. The tables summarize the endocytic proteins differentially enriched in WT (melanosome-like) and BAG6KO (exosome-like) B-16V EVs as analyzed using DAVID software and corresponding interaction networks generated by STRING are shown. Proteins in bold are deregulated in EVs but not in the corresponding cell lysate. Figure S2G shows validation of selected hits by western blotting. A list comparing EV and whole cell lysate protein levels by mass spectrometry is provided in Figure S2H. Two-tailed, unpaired Student's t-tests: ns, not significant, *p < 0.05, **p < 0.01, ***p < 0.001; SEM, standard error of the mean; WT, wild-type.

Selected mRNAs encoding disease-related, tumor-promoting factors that were enriched in BAG6KO-EVs were analyzed by qRT-PCR (Figure 2A). This analysis confirmed a higher abundance of these mRNAs in BAG6KO-EVs than in WT-EVs e.g. for *Ctnnal1* (encoding a-catulin, Catenin alpha-like protein 1), which is known to contribute critically to the invasive behavior of metastatic cells 22,23. Collectively, these data indicate that the presence of BAG6 in the EV-releasing cell impacts on the EV mRNA cargo.

Next, mass spectrometry (MS) was applied to comprehensively characterize the EV proteome under hypoxic conditions. As depicted in a volcano plot and a heat map (Figure 2B,C), 315 of about 3000 detected proteins were significantly (p<0.01) up- or downregulated and 33 were only detectable in either WT or BAG6KO samples (among these was BAG6 as expected) (MS data see Table S2).

Of note, differentially expressed proteins, either decreased (subcluster WT up) or increased (subcluster BAG6KO up) in BAG6KO-EVs clustered into distinct endosomal vesicle signatures (DAVID, functional annotation clustering; https://string-db.org(24)) (Figure 2B,C). In these subclusters, 12 out of 28 proteins (43%) were specifically upregulated in WT-EVs (but not in WT compared to BAG6KO whole cell lysates), while 22 out of 32 proteins (69%) were specifically enriched in BAG6KO-EVs (but not in corresponding whole cell lysates) (Figure S2F-H). This refers to a regulated EV cargo loading pathway dependent on BAG6 instead of an unspecific enrichment of cellular proteins in EVs.

WT-EVs-associated proteins such as RAB27, MREG, MYO5a or MYO7a are indicative for specialized vesicles, termed melanosomes, which are released by differentiated melanocytes 25, whereas these proteins were less abundant or not detectable in BAG6KO-EVs. Instead, specific proteins of the ESCRT complex, including ESCRT-I (TSG101), ESCRT-II (SNF8), as well as the ESCRT-III (CHMP2a, CHMP2b, CHMP4b and CHMP5) were significantly upregulated in BAG6KO-EVs. These proteins were previously identified in association with bona fide exosomes isolated from human dendritic cells 26 (Figure 2C).

Finally, further analysis revealed that proteins enriched in BAG6KO-EVs clustered into the processes of migration and angiogenesis. Upregulated angiogenesis-related proteins included SEMA3C, EDIL3, ADCY9, SRC, PRKCA, and SPHK1. On the contrary, TIMP3, an inhibitor of metalloproteinases involved in the re-modelling of the extracellular matrix via targeting ADAM10, ADAMts1 and ADAMts4 27 and thereby counteracting metastasis was significantly higher in WT-EVs compared to BAG6KO-EVs.

Thus, RNAseq and MS analysis revealed that both WT-EVs and BAG6KO-EVs originate from the endosomal compartment but represent distinct EV subtypes, probably generated via a BAG6-dependent pathway.

### BAG6-presenting but not BAG6-deficient EVs inhibit metastasis to the lung

**Figure 3:**
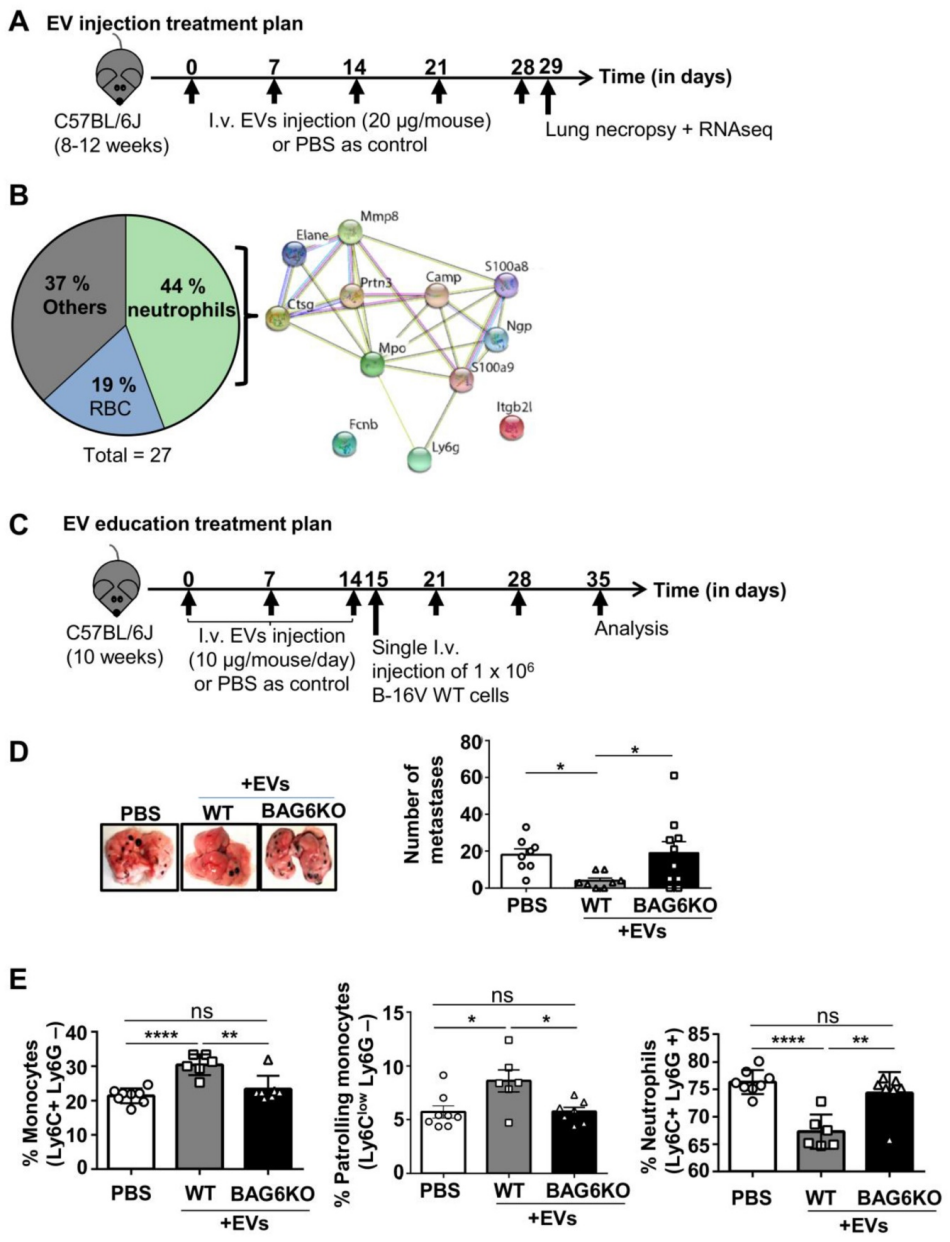
BAG6-presenting but not BAG6-deficient EVs inhibit metastasis to the lung. (A) Experimental treatment plan of EV injections (tumor cells were not injected). (B) Pie chart illustrating the enrichment analysis of RNAs upregulated in lungs of mice treated with BAG6KO B-16V EVs as compared to treatment with WT B-16V EVs or PBS (treatment plan provided in (A) with n=3 mice per group. The protein-protein interaction network of neutrophil-associated hits was constructed by STRING. Only two proteins did not show a direct functional interaction within this network, i.e. neutrophil integrin beta 2 like protein (Itgb2), which interacts with the extracellular matrix 64 and ficolin-B (FcnB), a neutrophil enriched lectin detecting pathogen associated molecular pattern (PAMPs) 65. A table summarizing all hits is provided in Figure S3A. (C) Experimental treatment plan of EV education prior to i.v. injection of B-16V cells to form experimental metastases. (D) Representative macroscopic appearance of lung metastases and quantification of lung metastases formed by B-16V WT cells intravenously injected into the tail vein of WT BJ/6 mice either educated with WT-EVs, BAG6KO-EVs or treated with PBS as control according to the treatment plan provided in (C). n=8 mice (PBS control), n=8 mice (injected with WT-EVs), n=10 mice (injected with BAG6KO-EVs). Statistical analysis was performed using non-parametric Kruskal-Wallis test (mean ranks compared with WT-EVs group) and Dunn's multiple comparisons test. Data are shown as mean ± SEM. (E) Flow cytometric analysis of isolated bone marrow cells, gated on CD11b+ cells and stained for neutrophils (Ly6C+ Ly6G+) and monocytes (Ly6C+ Ly6G- and Ly6C^low^ Ly6G-) from educated mice according to the treatment plan in (C) (analysis of n=8 mice (PBS control), n=6 mice (injected with WT-EVs), n=7 mice (injected with BAG6KO-EVs)). Statistical analysis was performed using non-parametric Kruskal-Wallis test (mean ranks compared with WT-EVs group) and Dunn's multiple comparisons test. Data are shown as mean ± SEM. The gating strategy of myeloid cells is provided in Figure S3E. ns, not significant, *p < 0.05, **p < 0.01, ***p < 0.001, ****p<0.0001; SEM, standard error of the mean; EVs, extracellular vesicles; i.v., intravenously injected; WT, wild-type; kDa, kilodalton; RBC, red blood cells.

Given the differences in mRNA and protein cargo loading of EVs isolated from WT and BAG6KO cells, we analyzed the *in vivo* activity of these EVs using an experimental B-16V lung metastasis model. Initially, WT-EVs or BAG6KO-EVs (20 μg, i.v., tail vein, protein/particle ratio see Figure S3B) or PBS (ctrl) were injected into tumor free *C57BL/6* mice weekly over a five-week period (Figure 3A). 24 hours following the last injection, the lung tissue was isolated and subjected to next generation RNA sequencing.

A significant upregulation of 50 transcripts was observed in the lungs from animals treated with WT-EVs compared to untreated controls and among these 6 transcripts were also significantly upregulated compared to BAG6KO-EV treated animals (Table S3A). These enriched mRNAs were involved in immunological defense pathways (DAVID, functional annotation clustering) and included the transcription factor *Spic*, which controls the development of red bone marrow and pulp macrophages. In line, the macrophage soluble defense receptor *Cd5l;* the interleukin *IL10;* the chemokine *CxCl13*, which is chemotactic for B cells and weakly for monocytes and macrophages and the Fc receptor-like scavenger receptor *Fcrls* were significantly increased. Secondly, transcripts for the NK cell lectin receptors *Klri1* and *Klrc3* were significantly higher expressed in lungs of WT-EVs-treated animals compared to PBS controls, although the comparison to BAG6KO-EV treatment did not reach significance (Figure S3A and Table S3A).

The analysis of animals treated with BAG6KO-EVs showed a significant upregulation of 27 transcripts in comparison to both untreated and WT-EVs-treated animals (Supplementary Table S3B). Most strikingly, almost half of the upregulated transcripts were indicative for the accumulation and activity of neutrophils (Figure 3B and Table S3B). Neutrophils are known to promote metastasis formation before cancer cell arrival 28, 29, although anti-tumor activity was obeserved in a immunodeficient model 15. Depicting these hits in an interaction enrichment network (https://string-db.orgstring) confirmed specific and functionally meaningful interactions (PPI enrichment p value of 1x10^-16^) (Figure 3B). The upregulation of selected neutrophil-associated hits (*S100a9, Elane, MPO, Prtn3*) was confirmed by qRT-PCR and immunohistochemistry (Figure S3C-D).

Taken together, EVs derived from WT or BAG6KO cells caused distinct gene expression changes in mouse lungs either corresponding to an anti- or pro-tumorigenic phenotype.

To investigate the impact of EVs on lung metastasis, animals were pre-treated with either WT-EVs or BAG6KO-EVs (10 µg every day for 14 days) or injected with PBS followed by the i.v. injection of wildtype B-16V tumor cells (Figure 3C).

EVs from WT cells inhibited lung metastasis significantly compared to untreated animals (Figure 3D). The inhibition of metastasis correlated with the accumulation of bone marrow monocytes, which corresponded to the Ly6C^low^ phenotype of anti-tumor patrolling monocytes 19 and a decrease of metastasis-promoting neutrophils in the bone marrow (Figure 3E, gating strategy presented in Figure S3E).

Surprisingly, treatment with BAG6KO-EVs had no significant effect compared with untreated controls. An increase of metastasis was not observed, although BAG6KO-EVs induced a pre-metastatic niche phenotype in the lungs (Figure 3B). This might be due to an interference of BAG6KO-EVs with WT-EVs released from the injected WT B-16V melanoma cells (which were not present in the experiment presented in Figure 3A,B).

TIMP3 was identified as a top hit among the proteins specifically enriched in WT-EVs (Table S3A) and therefore a candidate to execute the anti-metastatic function of WT-EVs compared to BAG6KO-EVs. TIMP3 is an inhibitor of the matrix metalloproteinases involved in migration, degradation of the extracellular matrix and macrophage polarization 27. Human cancers including melanoma show consistently that a high TIMP3 expression level is associated with a favourable patient survival (proteinatlas.org). B-16V melanoma cells incubated with BAG6KO-EVs exhibited an increase in wound closure as compared to WT-EV incubation and the PBS control (Figure S3F), consistent with the pro-tumor characterisation of the EV content (Figure 2A). This phenotype was reversed by overexpression of TIMP3, which is in line with a previous study showing that TIMP3 suppresses melanoma cell migration 31. In addition, *in vitro* differentiation of bone marrow-derived monocytes in the presence of WT-EVs polarized towards an inflammatory M1 macrophage phenotype characterised by increased mRNA expression of IL-12, Nos2 and Cxcl10 and characteristic dendritic morphology (Figure S3G). This phenotype was abrogated by incubation with BAG6KO-EVs and rescued by overexpression of TIMP3. These results suggest that the anti-metastatic activity might be at least partially mediated through BAG6-dependent recruitment of functional TIMP3 to EVs.

### p53 is critically involved in the inducible release of BAG6-presenting EVs

**Figure 4:**
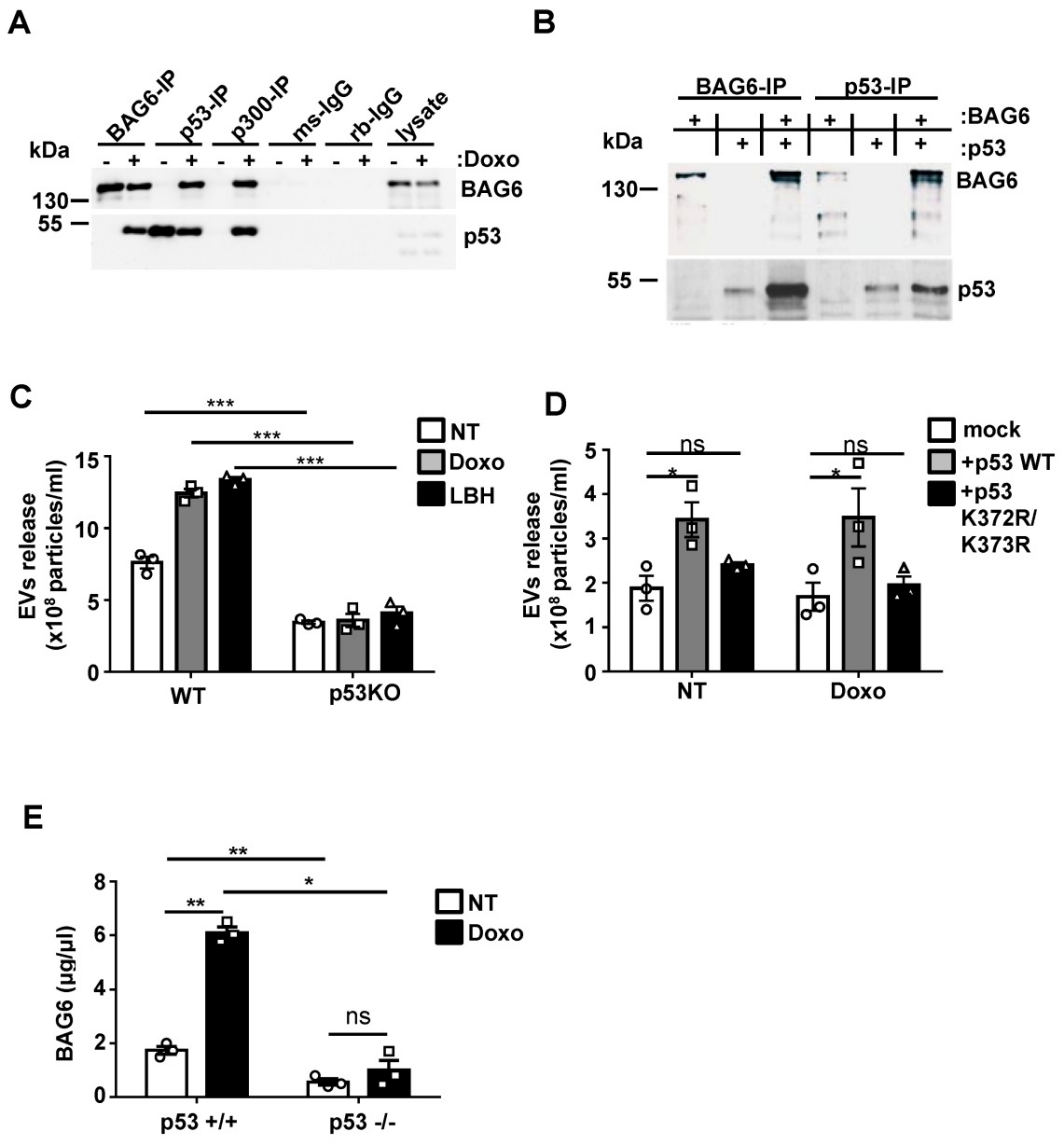
p53 is critically involved in the inducible release of BAG6-presenting EVs. (A) Immunoblot analysis of BAG6 and p53 in HEK293 cell lysates immunoprecipitated with anti-BAG6, anti-p53 or anti-p300 specific antibodies after treatment with 1 µM doxo for 1h or left untreated. Immunoprecipitation with either mouse (ms) or rabbit (rb) IgG isotype controls was performed as control and cell lysate was loaded as the input. Data are representative of 3 three independent experiments. The same experiment was conducted using HEK293 BAG6KO cells as a control (Figure S4A). HEK293 cells that were used to analyze the physical interaction of p53 with BAG6 and CBP/p300 are Ad-transformed cells and contain accumulated active p53(66) which is not further stabilized in reponse to doxo treatment as observed for B-16V (Figure S4D). (B) Immunoblot analysis of *in vitro* translated BAG6 and p53 mixed as indicated and incubated for 1h at 25° followed by precipitation using anti-BAG6 or anti-p53 antibodies. Data are representative of two independent experiments. (C) NTA analysis of EVs derived from HCT116 WT and p53-deficient cells that were either non-treated or treated with 100nM doxo or LBH for 16 hours. Particle measurements were normalized to protein concentration of corresponding total cell lysates and graphs represent mean ± SEM of three independent experiments. (D) NTA analysis of EVs derived from HCT116 p53-deficient cells that were either mock transfected or re-transfected with either WT p53 or a K373R/K373R acetylation-site mutant of p53 (p53 K372R/K373R) and non-treated or treated with 100nM doxo for 16h. Immunoblot analysis of the p53 reconstitution is provided in Figure S4C. (E) Detection of BAG6-associated EVs that were isolated from Eµ:Tcl1a;Cd19:Cre;Tp53 WT (p53^+/+^) or Eµ:Tcl1a;Cd19:Cre;Tp53fl/fl (p53^-/-^) mouse splenocytes (n=5) and either non-treated or treated *ex vivo* for 16h with 100nM doxorubicin using a BAG6-specific ELISA. Values were normalized to total protein amount determined by BCA assay. Two-tailed, unpaired Student's t-tests: ns, not significant, *p < 0.05, **p < 0.01, ***p < 0.001; SEM, standard error of the mean; EVs, extracellular vesicles; NT, non-treated; doxo, doxorubicin; LBH, LBH-589/Panobinostat; WT, wild-type; kDa, kilodalton; IP, immunoprecipitation; ms, mouse; rb, rabbit; IgG, immunoglobulin G.

The next set of experiments was performed to further analyze the molecular mechanisms responsible for distinct cargo loading and thus functional differences between WT- and BAG6KO-EVs. The release of BAG6-positive EVs could be induced by the inhibition of cellular de-acetylases (Figure 1A), suggesting that acetylation might be involved in the secretion. It is known that BAG6 is an essential co-enzyme for the major CBP/p300 acetyltransferase, first reported for the acetylation of p53 in a mouse model 32. This prompted us to test whether a BAG6/CBP/p300 complex formation was involved in the regulation of EV release. Immunoprecipitation (IP) of BAG6 from HEK293 cell lysates treated with doxo allowed the co-precipitation of p53 and vice versa (Figure 4A). Moreover, CBP/p300 antibodies co-precipitated both BAG6 and p53, suggesting that an acetyltransferase complex was formed upon doxo treatment (Figure 4A). IPs with BAG6-deficient cells confirmed the specificity of the antibodies used (Figure S4A). The interaction of *in vitro* translated proteins obtained in a cell free system confirmed binding of p53 and BAG6 (Figure 4B). Speculating that p53 and its acetylation in response to DNA damage was crucial for the inducible release of BAG6-presenting EVs, we tested the basal and inducible release from HCT116 cells and their p53-deficient (p53KO) derivative. As expected, p53KO cells failed to release increased amounts of EVs in response to both doxo- or LBH-treatment (Figure 4C). This phenotype could be rescued with p53WT but not with an acetylation-deficient p53 mutant (p53K372R/K373Rp53lys^372/lys373^) indicating that acetylation of p53 is indispensable for the EV release (Figure 4D; Figure S4C). Similarly, the inducible release of BAG6-positive EVs upon doxo treatment was abrogated in HEK293 cells with a siRNA-mediated p53 knock down (Figure S4B). To verify the *in vivo* relevance of this pathway, p53WT or p53KO splenocytes isolated from the established *Tcl1*-driven CLL mouse model were used 33. In line with the *in vitro* data, an induction of the secretion of BAG6-positive particles was restricted to p53WT cells (Figure 4E) confirming the impact of p53 on EV release.

### BAG6 and CBP/p300 are crucial for the acetylation of p53 and regulate the release of EVs

**Figure 5:**
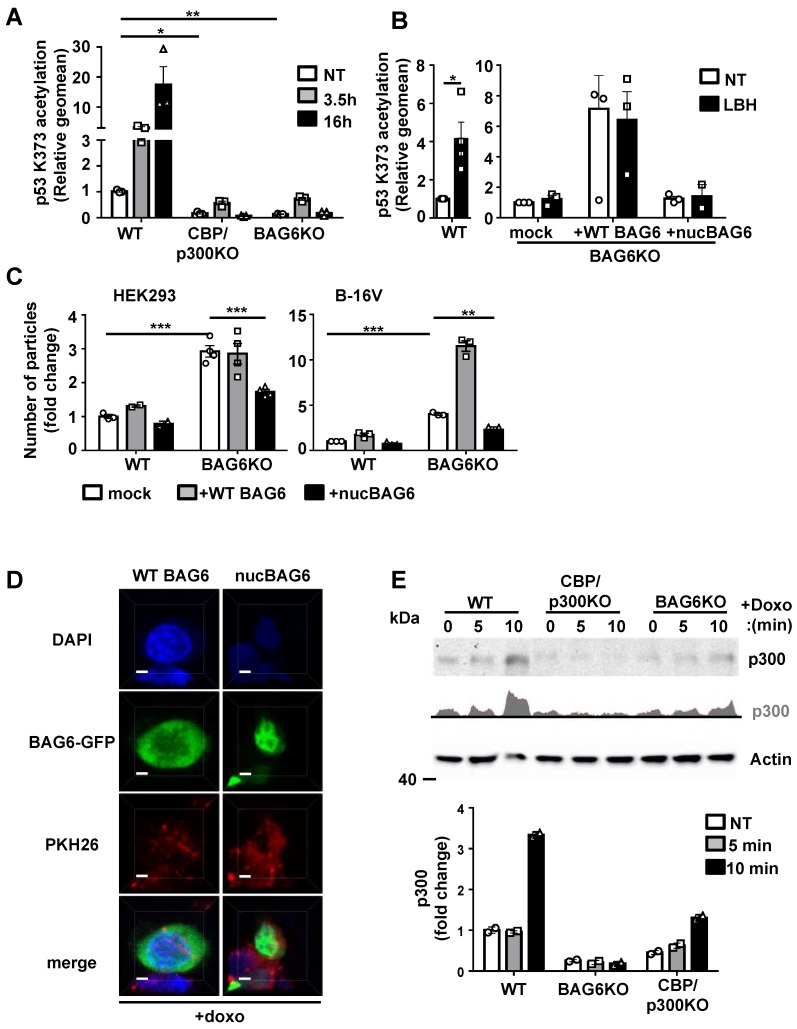
BAG6 and CBP/p300 are crucial for the acetylation of p53 and regulate the release of EVs. (A) Intracellular flow cytometric analysis of p53-acetylation (K373) in WT, BAG6KO or CBP/p300dKO HEK293 cells either non-treated or treated with 100 nM LBH for the indicated time. Bar graphs represent mean ± SEM of three independent experiments. A specific anti-p53K373 antibody and Dylight^TM^ 649 secondary antibody were used. (B) Intracellular flow cytometric analysis of p53-acetylation (K373) in BAG6KO HEK293 cells either non-treated or treated with 100 nM LBH for 16h upon mock-transfection or transfection with full-length BAG6 (+ WT BAG6) or with a N-terminal deleted BAG6 mutant (+nucBAG6). Bar graphs represent mean ± SEM of three independent experiments. A specific anti-p53K373 antibody and Dylight^TM^ 649 secondary antibody was used. (C) NTA analysis of 24h EV release from WT or BAG6KO HEK293 and B-16V cells that were either mock-transfected, transfected with full-length BAG6 (+WT BAG6) or with an N-terminal deleted BAG6 mutant (+nucBAG6). An immunoblot of the myc-tag transfected BAG6 is shown Figure S5A. A cartoon illustrating the impact of BAG6 expression and subcellular localization on the EV release is shown in Figure S5D. (D) Immunofluorescence microscopic analysis of HEK293 cells transfected with either full length BAG6 (+WT BAG6) or N-terminal deleted BAG6 mutant (+nucBAG6) and treated with 1 µM doxo for 1h. Transfected BAG6 was visualized by a GFP-tag, the nucleus and cell membrane were visualized by staining with DAPI and PKH, respectively, and merged images are shown. Scale bar: 2 µm. The analoguous experiment using non-treated HEK293 cells is shown in Figure S5B. (E) Immunoblot analysis of CBP/p300 in cytoplasmic fractions extracted from WT, BAG6KO and CBP/p300dKO-HEK293 cells that were either non-treated or treated with 1µM Doxo for the indicated time. The bar graph represents quantification of two independent immunoblots from biological replicates normalized to actin as the loading control. Two-tailed, unpaired Student's t-tests: *p < 0.05, **p < 0.01, ***p < 0.001; SEM, standard error of the mean; NT, non-treated; WT, wild-type; EV, extracellular vesicles; doxo, doxorubicin; kDa, kilodalton; AC, acetylation; DAPI, 4′,6-Diamidin-2-phenylindol, GFP, green fluorescent protein.

To address the impact of BAG6/CBP/p300 for p53-acetylation and subsequent EV release, we used HEK293 and B-16V BAG6KO and CBP/p300 double KO (dKO) cells (characterization of CBP/p300 dKO cell lines 34. As expected, dKO of the acetyltransferases CBP/p300 abrogated the acetylation of p53 at Lysine 373 (p53K373) nearly completely and the depletion of BAG6 affected this acetylation in a similar manner (Figure 5A) confirming that BAG6 was crucial for p53K373 acetylation. The p53-acetylation could be restored upon re-expression of WT BAG6 in BAG6KO cells, which was not observed upon transfection of an N-terminal deleted BAG6 mutant (constitutive nuclear mutant, nucBAG6 aa555-1132) (Figure 5B and S5A,B). Subsequently, the release of EVs from human HEK293 and mouse B-16V BAG6 cells was analyzed. Surprisingly, we observed a significantly higher basal release of EVs in the BAG6KO cell line compared to their WT parental cells, indicating that BAG6 inhibited EV release under non-stress conditions (Figure 5C, white bars).

This phenotype could be rescued in both BAG6KO cell lines upon expression of an N-terminal BAG6-deletion mutant (nucBAG6), which reduced the EV release significantly (Figure 5C, black bars). The nucBAG6 mutant is strictly nuclear and, unlike the WT BAG6 protein, does not translocate to the cytoplasm e.g. upon doxo treatment 15, 35 (Figure 5D and S5B,C).

In line, the overexpression of WT BAG6, which is able to shuttle between the nucleus and the cytoplasm, did not reduce the EV release (Figure 5C, grey bars and cartoon in Figure S5D).

To account for the possibility of differences in EV release due to single cell clones heterogeneity, we have generated several WT and BAG6KO B-16V clones (Figure S2A) and consistently observed an increase in EV release from BAG6KO cell clones compared to WT clones supporting a mechanistic rather than stochastic regulation.

BAG6 was previously identified as a nuclear-cytoplasma shuttle for CBP/p300 35. To investigate whether the inhibition of the EV release by nuclear BAG6 was associated with the capture of CBP/p300 in the nucleus, we analyzed the CBP/p300 translocation in HEK293 WT and BAG6KO cells. Western blots of cytoplasmic HEK293 cell fractions revealed an increase of cytoplasmic CBP/p300 upon doxo treatment, which was diminished in BAG6KO cells (Figure 5E).

These results suggest that the BAG6/CBP/p300 acetyltransferase complex directs the release of EVs. Under basal conditions BAG6 localized predominantly in the nucleus and the release of EVs was low. Doxo treatment induced the nuclear export of BAG6 and CBP/p300 to allow the acetylation of p53, which triggered the release of BAG6-positive exosomes with anti-metastatic activity. Depletion of BAG6 abolished the inhibitory activity of nuclear BAG6 and allowed an increased release of BAG6-negative EVs with pro-tumorigenic cargo (Figure 5C), which in turn was blocked by overexpression of a nuclear BAG6 mutant unable to shuttle to the cytoplasm.

### Doxo treatment induces complex formation between BAG6 and ESCRT proteins

We next addressed the molecular link of the BAG6-acetylation complex and EV release. The best-studied pathway involved in EV biogenesis and secretion is the ESCRT pathway formed by four complexes, ESCRT-0, ESCRT-I, ESCRT-II, and ESCRT-III acting successively and in concert with accessory proteins 36.

**Figure 6:**
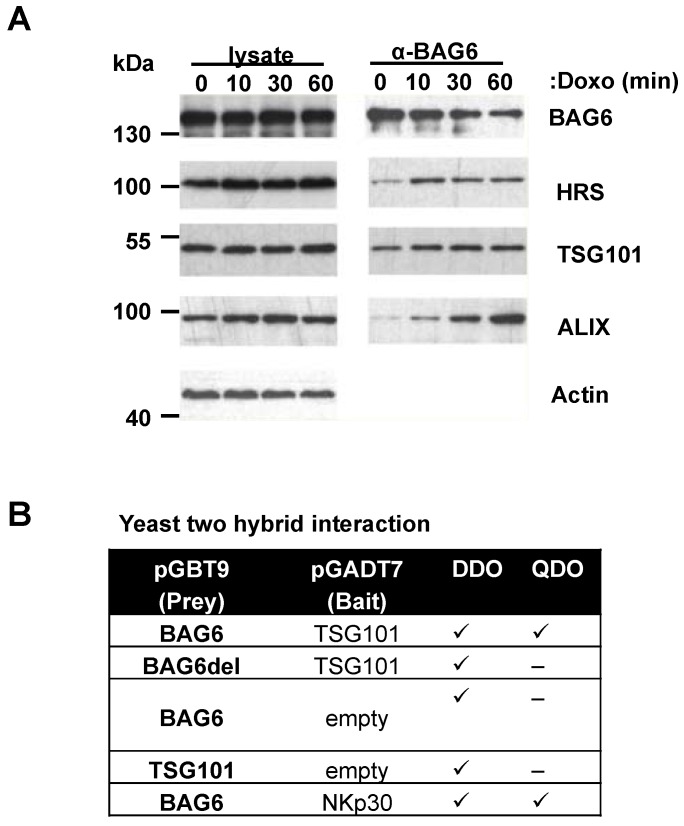
Doxo treatment induces complex formation between BAG6 and ESCRT proteins. (A) Immunoblot analysis of cell lysates and BAG6 immunoprecipitations of HEK293 cells that were either non-treated or treated with 1µM doxorubicin for the indicated time probed with specific antibodies against BAG6, the ESCRT proteins HRS, TSG101 and ALIX and actin as a loading control. The blot represents one out of two independent experiments. (B) Analysis of the interaction of BAG6 and TSG101 using the Yeast two hybrid method. BAG6, BAG6 without TSG101 binding motif (BAG6del with deleted PSAP/PTAP motifs at amino acid positions 471-475 and 496-499, see Figure S6A) and TSG101 were subcloned in pGADT7 and pGBT9 expression vectors transformed into yeast lines Y2H Gold and Y187 or control vectors and mated. NKp30 and BAG6 were used as a positive control for interaction analysis and experiments were performed in two independent runs. doxo, doxorubicin; kDa, kilodalton; DDO, Minimal Media Double Dropouts; QDO, Minimal Media Quadruple Dropouts (QDO)

Co-immunoprecipitation experiments revelead an association of BAG6 with endogenous ESCRT-proteins HRS, TSG101 and Alix and this interaction was increased in response to doxo treatment (Figure 6A). In line, sequence analysis of the BAG6 gene revealed a late endosomal motif P(S/T)AP within the sequence, which is known to recruit TSG101 (Figure S6A). Late endosomal motifs LYPX_n_L and the P(S/T)AP motif were initially discovered in HIV-1 (human immunodeficiency virus) and EIAV (equine infectious anaemia virus) and are required for viral recruitment of the ESCRT-complex to ensure the efficient release of nascent virions from the infected cells 37. Direct binding of BAG6 to TSG101 was in fact mediated and dependent on this P(S/T)AP motif as shown by yeast two hybrid experiments with a WT BAG6 and a PSAP/PTAP-deletion mutant, respectively (Figure 6B). Altogether, these experiments suggest the involvement of the ESCRT complex in the BAG6-mediated EV release.

### Relevance of BAG6-dependent EV formation in human melanoma

**Figure 7:**
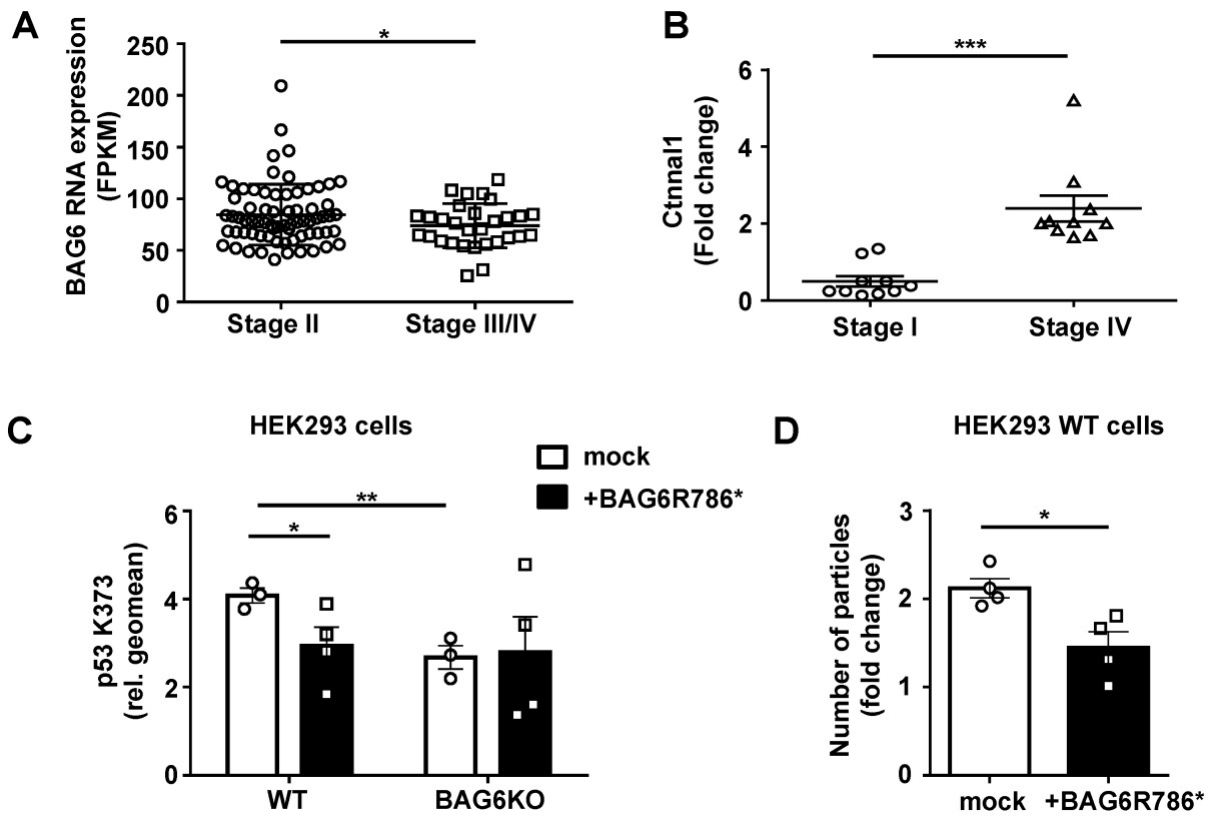
Relevance of BAG6-dependent EV formation in human melanoma. (A) TCGA data analysis comparing BAG6 RNA tissue expression level of stage II (n=71) versus stage III/IV (n=29) melanoma patients. * indicates p = 0.0462 of unpaired t-test with Welch's correction. (B) qRT-PCR analysis of Ctnnal1 in EVs isolated from the plasma of melanoma patients comparing stage I and stage IV disease. The graph depicts the comparison of Ctnnal1 expression levels as fold change between stage I (n=10) and stage IV (n=10) patients. * indicates p = 0.0002 of unpaired t-test with Welch's correction. A control experiment using cDNA made from isolated patient plasma EV-RNA is shown in Figure S7A. (C) Intracellular flow cytometric analysis of p53-acetylation (K373) depicted for WT or BAG6KO-HEK293 cells either mock-transfected or transfected with the BAG6R786* mutant and 24h later treated with 100 nM LBH for 16h. Bar graphs represent mean ± SEM of three independent experiments. Statistical analysis was performed using two-tailed, unpaired Student's t-tests. A schematic and an immunoblot showing the expression of the BAG6R786* mutant protein is shown in Figure S7B and S7C, respectively. (D) NTA analysis of 16h EV release from WT HEK293 cells that were either mock-transfected or transfected with the BAG6R786* mutant 24h prior to the treatment with 100 nM LBH during EV collection. Bar graphs represent mean ± SEM of four independent experiments. Statistical analysis was performed using two-tailed, unpaired Student's t-tests. *p < 0.05, **p < 0.01, ***p < 0.001; SEM, standard error of the mean; WT, wild-type; BAG6R786, BAG6 mutant with stop codon at Arginin position 786.

Based on our *in vitro* and *in vivo* mouse data, we speculate that late, metastatic melanoma stages are characterized by the presence of pro-metastatic EVs resembling BAG6KO-EVs isolated from B-16V BAG6KO cells. Consistent with this hypothesis, analysis of TCGA data deposited at proteinatlas.org (/ENSG00000204463-BAG6/pathology/tissue/ melanoma#12000001big) showed that the expression of *BAG6* declined in melanomas with tumor progression and was significantly lower in metastatic stages III/IV compared to early stages (Figure 7A). Moreover, transcripts for the oncoprotein CTNNAL1 (α-Catulin) which is known to drive malignant invasion and metastasis 22, 23 were clearly detectable and significantly enriched in EVs from the plasma of metastatic late stage patients compared to EVs from stage I patients (Figure 7B). A similar phenotype was observed in EVs collected from BAG6KO mouse cells (Figure 2A).

Mutations in the *BAG6* gene, which we consider a novel tumor suppessor gene, are rarely detected in melanoma and other tumors, but we investigated a specific mutation described in a melanoma and a pancreatic cancer patient (uniprot/BAG6_HUMAN, stop gained BAG6 R786*). This mutation at codon 786 generates a stop codon and resulted in the expression of a truncated protein upon transfection (Figure S7B and S7C). BAG6R786* was unable to rescue the p53K373 acetylation null phenotype upon transfection in BAG6KO cells. Instead it diminished the p53-acetylation in WT BAG6 cells significantly (Figure 7C) and can thus be considered as a dominant negative mutant of BAG6, which is known to form dimers 38 (control experiment in Figure S7A). In line with our finding of the requirement for p53 acetylation in EV release (Figure 4), transfection of BAG6R786* resulted in a reduced number of particles secreted by WT cells (Figure 7D). Taken together, these data suggest that a diminished expression or defects in the activity of BAG6 impact on the formation and release of anti- versus pro-metastatic EVs.

## Discussion

Recent research on tumor cell-released, and specifically on melanoma cell-derived EVs, highlight their crucial role for tumor progression and for the formation of pre-metastatic niches 39-41. On the contrary, several studies reported the existence of EVs promoting melanoma tumor cell clearance predominantly via immune cell activation 7,8. However, little is known about the mechanisms that are involved in EV formation and cargo loading, which regulate distinct functions of EVs. Here we present evidence that the chaperone BAG6 is a molecular switch directing the release of EVs with either anti- or pro-metastatic activity (see summary model, Figure S9).

This is initially based on the finding that EVs from BAG6-proficient and BAG6-deficient melanoma cells were characterized by distinct protein and mRNA cargo loading (Figure 2). Both EV types changed the gene expression profile in the lungs of mice (Figure 3), which is compatible with the detected similar level of integrin-6, known to be associated with an exosomal tropism to the lung 42. However, we observed distinct molecular changes in the lungs of mice conditioned with these specific EV subtypes reflecting either anti- or pro-metastatic activity. WT-EVs not only induced the expression of genes with reported anti-tumor activity but also caused accumulation of patrolling Ly6C^low^ monocytes, which are known to cause tumor clearance at the metastatic niche in a NK cell-dependent manner 8, 19. Moreover, EVs collected from the B-16V WT cells suppressed lung metastasis whereas EVs from BAG6KO cells failed to inhibit lung metastasis in the B-16V experimental transplantation model (Figure 3). In contrast, BAG6KO-EVs induced a neutrophil gene signature indicative for the formation of a pre-metastatic niche 20. Recently, the overexpression of the serine protease inhibitor and tumor suppressor PEDF (although not detectable in the murine B-16V EVs) was shown to turn metastasis-promoting EVs into tumor-inhibiting melanoma EVs 8. Notably, the targets of PEDF, elane (elastase) and cathepsin G, which are indispensible for melanoma metastasis 43 were both significantly induced upon B-16V BAG6KO-EVs treatment in mouse lungs (but not in response to WT-EVs). We observed direct uptake of B-16V EVs by neutrophils in the lung of reporter mice using the Cre-loxP method of EV transfer (Figure S8, 44 and expect that effects mediated by WT- and BAG6KO-EVs might be both direct and indirect as observed in other studies 45.

Analyzing the top hit enriched in WT-EVs, we identified TIMP3 as one of the strong candidates, which might contribute to their anti-metastatic activity (Figure S3F,G). However, it seems unlikely that the anti-metastatic activity is mediated exclusively by the action of a single candidate molecule (such as BAG6 or TIMP3), but is rather determined by the interplay of the specific EV cargo composition defined by the biogenesis pathway.

The EV content and immune-alert activity changes in response to cellular stress stimuli including doxorubicin, HDAC-inhibition and hypoxia (this study), receptor activation (RIGI) or cytokines (IFNg). Here we established a direct molecular link of BAG6 to the formation of such stress-induced EVs. Mechanistically, the release required the concerted and inducible translocation of BAG6 and CBP/p300 into the cytoplasm and the acetylation of p53 (Figure 4 and 5). The acetyltransferase cofactor activity of BAG6 regulating CBP/p300 is involved in different cellular processes including metabolic regulation 46 or apoptosis 32. Recently, a role for BAG6 and p300-dependent acetylation in autophagy was reported 35. Nuclear import of BAG6 and p300 in response to starvation allowed the acetylation of nuclear p53, which triggered autophagy, whereas basal cytoplasmic BAG6 level was sufficient to acetylate ATG7, in turn inhibiting this process. The nuclear-cytoplasmic shuttle of BAG6 together with the associated acetyltransferases might explain that autophagy and the release of exosomes are considered as mutually exclusive cellular responses 47. In line, cytoplasmic p53, but not nuclear p53 was shown to inhibit autophagy 48, although the underlying mechanisms are still discussed controversely 49.

A role for p53 in exosome secretion was already suggested 50 and recent work confirmed that p53 deficiency or mutations lead to the enrichment of tumor-promoting proteins in released EVs 51. In line with a link of BAG6 and p53 in EV biogenesis it was reported that p53 deficiency reduced the amount of the ESCRT component HRS (which interacts with BAG6, Figure 5) in exosomes of colon carcinoma cells. Notably, gene enrichment analysis using CPDB performed on the proteomic EV data of HCT116 p53null and wildtype cells showed that p53null EVs also up-regulated pathways associated with tumor progression (e.g. angiogenesis p=0.000352 and TGFβ p=0.00399). In addition, exosomes from p53null cells of this study were smaller in size compared with their WT counterparts, a phenotype reminiscent to the BAG6KO-EVs phenotype shown in our study (Figure 1). Interestingly, previous studies have also shown that different stress stimuli like heat shock and cisplatin treatment induced the release of EVs with smaller size based on EM measurements and these EVs exhibited enhanced ability to induce invasive behavior 52, 53. Furthermore, acidity, known to contribute to the immune escape of cancer cells 54,55 has been reported as another important stress factor within the tumour microenvironment to induce increased amounts of EVs from melanoma cells 56,57.

The ability of BAG6 to recruit to the exosomal pathway via association with components of the ESCRT-complex (Figure 6) may explain its regulatory role on EV cargo loading. We found the late endosomal motif P(S/T)AP of BAG6 to be important for the direct association with TSG101, an interaction mechanism reminiscent to the ESCRT recruitment during virus assembly 37. Recently, the recruitment of galectin-3 to exosomes was shown to be dependent on a P(S/T)AP motif 58 suggesting that this sequence can be regarded as an exosomal targeting sequence directing the packaging of soluble proteins in or on exosomes. Recently, BAG6 was reported to control the activity of Rab GTPases, which are known regulators of intracellular vesicle trafficking 59. Notably, the regulation of specific Rab GTPases is an attractive hypothesis to explain the surprising increase in EV release in the absence of cellular BAG6 under non-stress conditions and requires further investigations.

In view of the important role of BAG6 together with CBP/p300/p53 in tumor immune surveillance, an impaired function of BAG6 and CBP/p300 might be expected, as generally observed for p53. However, mutations in these genes in tumors are rather rare and defects might be related to posttranslational modifications or epigenetic aberrations. In fact, re-expression of BAG6 and CBP/p300 upon inhibition of the transcriptional repressor BCL6 rendered B cell lymphoma cells and xenografts susceptible to HDACi and HSP90 therapy 60. Tumor suppressor features for BAG6 were previously described and BAG6 polymorphisms were associated with lung cancer risk e.g. based on genome-wide association studies 61. In line with tumor-suppressing activity, we observed that a rare BAG6 mutation identified in a melanoma and pancreatic cancer patient encoding a truncated protein had dominant negative activity affecting the acetyltransferase activity of CBP/p300/BAG6 (Figure 7). Highlighting the importance of BAG6-regulated EVs, we detected (tumor-promoting) vesicular α-catulin mRNA (enriched in mouse BAG6KO-EVs compared to WT-EVs) exclusively in late stage EVs from metastatic melanoma patients, and this correlated inversely with the cellular BAG6 transcript expression. More extensive dissection of the BAG6/CBP/p300-p53 pathway, e.g. by stratifying tumor patients and their p53 status as well as analysis of additional cell and patient settings will be necessary in future studies. We hypothesize that the described pathway might be affected by p53 mutations, but might also be influenced by mutations in other stress-related pathways, for instance a deregulated Retinoblastoma pathway which is often observed in melanoma patients.

In conclusion, our findings identify BAG6 as a crucial regulator of immune alert, anti-metastatic EVs via BAG6/CBP/p300-mediated acetylation of p53 and direct association with the ESCRT machinery via its P(S/T)AP motif. Loss of BAG6 allowed the release of EVs with pro-tumorigenic cargo and activity. This renders BAG6 a potential therapeutic tool to specifically interfere with the biogenesis of exosomes, which can re-program the biological activity of tumor-cell derived EVs into anti-cancer moieties.

## Materials and Methods

### Human samples

Plasma was obtained with informed written consent by the patients and approval by the local ethics committee of the University Hospital Essen [ref. no. 11-4715].

### *In vivo* treatment experiments and sample preparation from experimental mice

8-12 weeks old C57BL/6J mice, housed and fed under pathogen-free conditions, were injected with EVs or cells intravenously into the tail vein. Dissected mouse lungs were either frozen in optimal-cutting-temperature compound (OCT Tissue Tec) on dry ice and stored at -80°C for immunohistochemistry or directly taken up in RNA stabilization solution and frozen at -20°C until further processed.

### Cell treatment

Cell lines were treated for 16 h with 100 nM doxorubicin or 100nM LBH, respectively. For short term (5- 120 min) DNA damage induction, cells were treated with 1 or 10 µM doxorubicin (as stated in the figure legends).

### Plasmids and transfection

pcDNA3.1 wild-type BAG6 (BAT3) and nucBAG6 are described (15). pcDNA3.1 BAG6R786* was generated by site-directed mutagenesis using QuickChange II Site-Directed Mutagenesis Kit and the expression plasmid pcDNA3.1 wild-type BAG6 as a template. HRS expression vector pCS2 was obtained from Addgene (#29685) and pcDNA3.1 for p53 was kindly provided by Dr. Pattingre (INSERM, France).

### Antibodies

All antibodies used are listed in Table S4.

### *In vitro* expression assay

*In vitro* expression experiments were performed using cell-free protein expression kit based on Leishmania tarentolae. Recombinant proteins were obtained by cloning in pLEXSY-invitro vector.

### Immunoprecipitation

Cell lysates or *in vitro* expressed proteins were precipitated using specific antibodies against BAG6, p53, CBP/p300, HRS, ubiquitin or acetyl-lysine. A minimum of 1 µg of antibody were used for 100 µg of total protein and Protein A magnetic beads were used for pull-down.

### EV preparation

EVs were collected in cell culture for either 24 or 48h in either EV-depleted medium or protein-free CD293 medium. For purifcation, consecutive pre-centrifugation steps at 300 x g (10 min), 2,000 x g (10 min) and 3,500 x g (20 min) for clearance of cells and cellular debris was performed before ultracentrifugation at 10,000 x g (60 min) and/or 100,000 x g (90 min) using SW 41 Ti rotor or Type 45Ti for at least two times with intermediate resuspension in PBS or HBSS and ultra-centrifugation at the respective g force using TLA-45 rotor in the last centrifugation. EVs were resuspended in PBS or HBSS. The amount of EV protein was quantified by Nanodrop 1000 and/or using BCA assay. The number of particles was determined by Nanoparticle Tracking Analysis.

### Quantitative RT-PCR

RNA from EVs or lung tissue was reverse transcribed with RevertAid First Strand cDNA Sysnthesis Kit using oligo-d(T) primers and/or random primers. Quantitative PCR measurements were performed on a 7500 real-time PCR system using SYBR Green. Initial heat inactivation was 95°C for 15 min and 40 cycles of 15 sec at 94°C, 30 sec at 56°C, and 30 sec at 72°C were performed followed by melting curve analysis. All primers used are listed in Table S5.

### Mass spectrometry bioinformatics and statistical analysis

All mass spectrometric raw data were processed with Maxquant using default parameters. Briefly, MS2 spectra were searched against the Uniprot MOUSE database, including a list of common contaminants. False discovery rates on protein and PSM level were estimated by the target-decoy approach to 1% (Protein FDR) and 1% (PSM FDR), respectively. The minimal peptide length was set to 7 amino acids and carbamidomethyolation at cysteine residues was considered as a fixed modification. Oxidation (M) was included as variable modification. The match-between runs option was enabled. LFQ quantification was enabled using default settings. Downstream data processing was conducted within the Perseus computational platform. Briefly, protein groups flagged as „reverse“, „potential contaminant“ or „only identified by site“ were removed from the data. LFQ data were log2 transformed. Statistical analysis of differentially regulated proteins was performed using a two-sided t-test (fudge factor s0 was adjusted to 0.1). Resulting p values were corrected for multiple testing using a permutation-based FDR approach.

### RNAseq

RNA quality was assessed using the Experion RNA StdSens Analysis Kit. RNA-seq libraries were prepared from total RNA using the TruSeq Stranded mRNA LT kit according to the manufacturer's instructions. Quality of sequencing libraries was controlled on a Bioanalyzer 2100 using the Agilent High Sensitivity DNA Kit. Pooled sequencing libraries were quantified with digital PCR (QuantStudio 3D) and sequenced on the HiSeq 1500 Illumina platform in Rapid-Run mode with 50 base single reads. RNAseq was performed from RNA isolated using the RNAeasy mini kit with the Illumina Truseq mRNA kit v2 on an Illumina Hiseq 1500 according to the manufacturer's instructions.

### RNA-Seq Bioinformatic Analysis

Raw transcriptome reads were aligned to the mouse genome from Ensembl 89 using STAR version 2.4.1a 62. Gene expression was quantified on gene models that included only protein coding transcripts for protein coding genes using custom python scripts. Differential genes were called using edgeR 63 at a threshold of FDR <= 0.05; |log2FC| >= 1 and counts per million >= .3.

### Statistical analysis

Statistical analyses were performed using Graphpad Prism software. Two-tailed, unpaired Student's t-tests were performed to analyze the significance of mean values between two variables, if not otherwise stated. Statistical analysis of EVs education animal experiments was performed using non-parametric Kruskal-Wallis test (mean ranks compared with WT-EVs group) and Dunn's multiple comparisons test. An unpaired Welch's t-test was performed to analyze TCGA data to account for unequal sample sizes.

### Data deposition

The mass spectrometry proteomics data have been deposited to the ProteomeXchange Consortium via the PRIDE partner repository with the dataset identifier PXD010677. RNAseq data of mouse lungs were deposited at ArrayExpress accession E-MTAB-7119. RNAseq data of B-16V EVs were deposited at ArrayExpress accession E-MTAB-7119.

## Supplementary Material

Supplementary figures, table S4-S5, materials and methods.

Supplementary table S1.

Supplementary table S2.

Supplementary table S3A.

Supplementary table S3B.
