# Supplementary material for: Exosome-dependent immune surveillance at the metastatic niche requires BAG6 and CBP/p300-dependent acetylation of p53
Source: Theranostics. 2019 Aug 14;9(21):6047–62. doi: 10.7150/thno.36378 (PMC6735508; doi:10.7150/thno.36378)
Supplement: Supplementary file 1 — Supplementary figures, table S4-S5, materials and methods. [file thnov09p6047s1.pdf]

SUPPLEMENTARY FIGURES

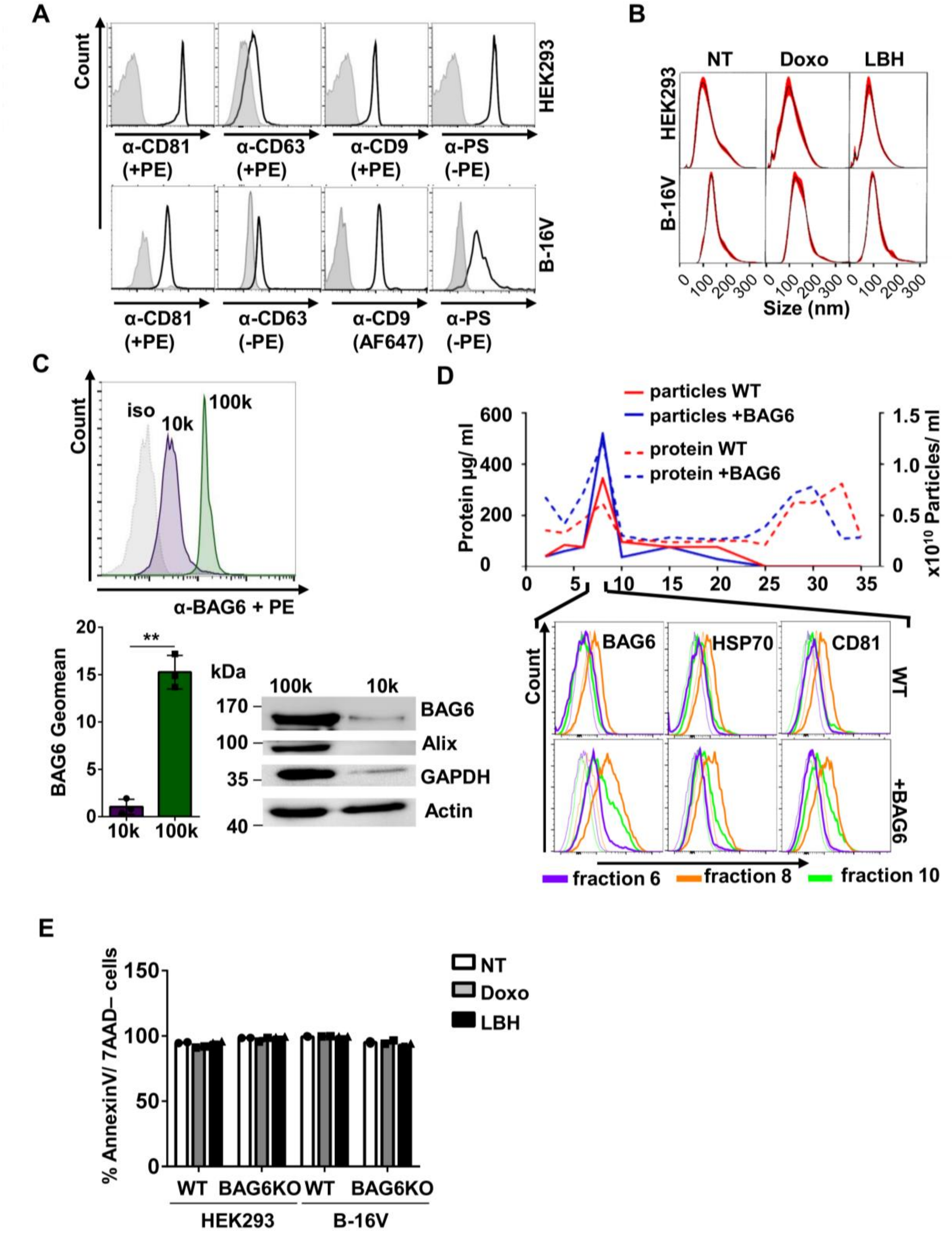

Figure S1. Supplement related to Figure 1.

(A) The presence of the EV-markers CD81, CD63, CD9 and phophatidylserine on purified particles isolated by ultracentrifugation from HEK293 and B-16V cell supernatants was verified using bead-assisted flow cytometry. The data is representative of three independent experiments. Specific antibodies were either directly labelled (-) or probed with a secondary fluorochrome-labelled antibody (+).

(B) Representative size distribution histograms obtained by NTA analysis of EVs isolated from HEK293 and B-16V cell supernatants either non-treated or treated with 100nM doxo or 100nM LBH for 16 hours. NTA confirmed that the purified EVs with or without treatment corresponded to small particles with a mean diameter of about 100-150nm, which are referred to as exosomes by many authors. The data are representative of three independent experiments.

(C) Analysis of BAG6 expression by bead-assisted flow cytometry and immunoblotting with indicated antibodies of HEK293 EVs purified by serial ultracentrifugation at 10k x g (larger EVs up to 1000µm) and 100k x g (exosome-like sized EVs) showing that BAG6 is predominantly associated with 100k x g fraction. The experiment was performed two independent times.

(D) Free flow electrophoresis (FFE) was applied to isolate EVs from HEK293 wild-type cells (WT) and BAG6 overexpressing cells (+BAG6) since the purification of EVs using ultracentrifugation cannot fully exclude the co-precipitation of contaminating soluble proteins. The plot shows the protein concentration by BCA assay and particle concentration by NTA analysis of the separated fractions. EVs associated with BAG6 and with the EV-markers HSP70 and CD81 were specifically detected by bead-assisted flow cytometry in the EV-containing fraction 8, but were absent in the soluble protein-rich fractions 25-33.

(E) Flow cytometric analysis of 7AAD and AnnexinV stainings of HEK293 and B-16V cells either non-treated, treated with 100nM doxo or with 100 nM LBH for 16h to ensure viability of the releasing cells (% AnnexinV/7AAD negative cells is depicted). Viability was checked regularly and data represent two independent experiments.

NT, non-treated; iso, isotype control antibody; 10k, ultracentrifugation fraction at 10k x g; 100k, ultracentrifugation fraction at 100k x g; WT, wild-type; kDa, kilodalton; doxo, doxorubicin; LBH, LBH-589/Panobinostat.

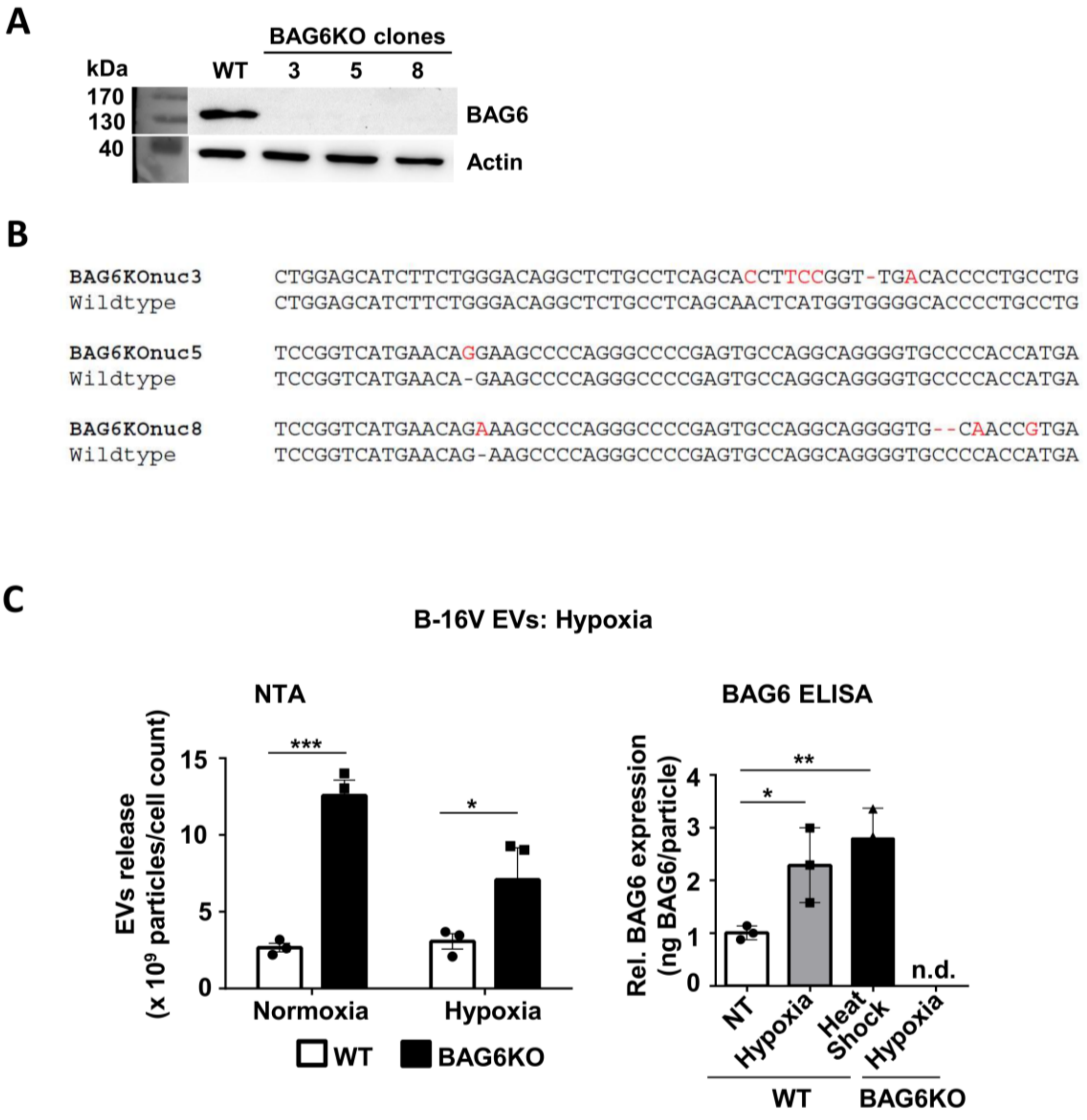
A
BAG6KO clones
kDa
WT
3
5
8
170
130
40
BAG6
Actin
B
BAG6KOnuc3
CTGGAGCATCTTCTGGGACAGGCTCTGCCTCAGCACCTTCCGGT-TGACACCCCTGCCTG
Wildtype
CTGGAGCATCTTCTGGGACAGGCTCTGCCTCAGCAACTCATGGTGGGGCACCCCTGCCTG
BAG6KOnuc5
TCCGGTCATGAACAGGAAGCCCCAGGGCCCCGAGTGCCAGGCAGGGGTGCCCCACCATGA
Wildtype
TCCGGTCATGAACA-GAAGCCCCAGGGCCCCGAGTGCCAGGCAGGGGTGCCCCACCATGA
BAG6KOnuc8
TCCGGTCATGAACAGAAAGCCCCAGGGCCCCGAGTGCCAGGCAGGGGTG--CAACCGTGA
Wildtype
TCCGGTCATGAACAG-AAGCCCCAGGGCCCCGAGTGCCAGGCAGGGGTGCCCCACCATGA
C
B-16V EVs: Hypoxia
NTA
EVs release
(x 10⁹ particles/cell count)
***
*
15
10
5
0
Normoxia
Hypoxia
WT
BAG6KO
BAG6 ELISA
Rel. BAG6 expression
(ng BAG6/particle)
**
*
4
3
2
1
0
n.d.
NT
Hypoxia
Heat Shock
Hypoxia
WT
BAG6KO

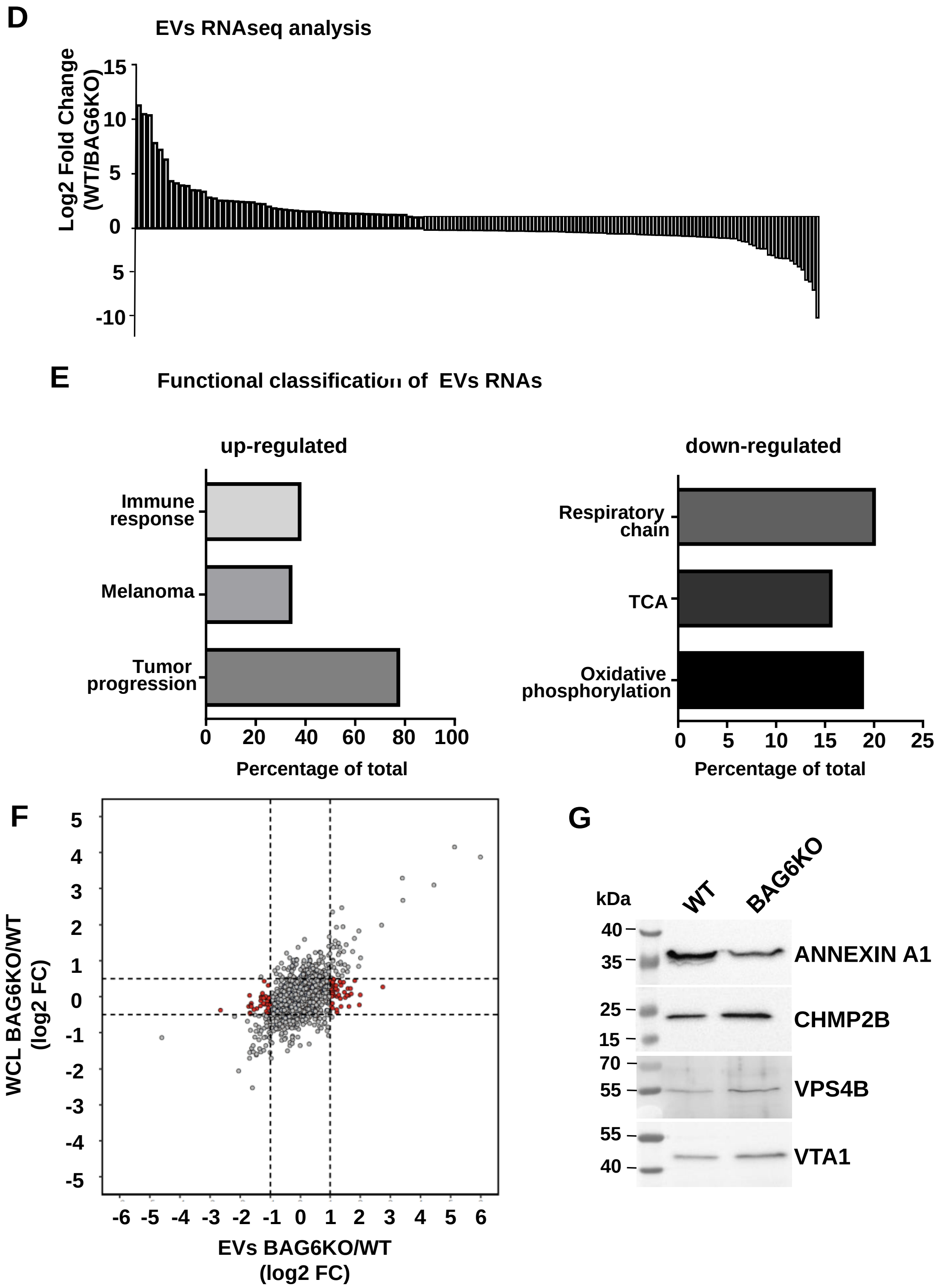
D
EVs RNAseq analysis
Log2 Fold Change (WT/BAG6KO)
15
10
5
0
5
-10
E
Functional classification of EVs RNAs
up-regulated
Immune response
Melanoma
Tumor progression
0 20 40 60 80 100
Percentage of total
down-regulated
Respiratory chain
TCA
Oxidative phosphorylation
0 5 10 15 20 25
Percentage of total
F
WCL BAG6KO/WT (log2 FC)
5 4 3 2 1 0 -1 -2 -3 -4 -5
-6 -5 -4 -3 -2 -1 0 1 2 3 4 5 6
EVs BAG6KO/WT (log2 FC)
G
kDa
WT
BAG6KO
40
35
ANNEXIN A1
25
15
CHMP2B
70
55
VPS4B
55
40
VTA1

H

Melanosome-like WT-EVs

		EV proteome		Cellular proteome	
	**Gene names**	**log2 FC BAG6KO/WT**	**q-value**	**log2 FC BAG6KO/WT**	**q-value**
Specifically deregulated in EVs	Cnp	-1,86	0,00E+00	N/A	1,00E+00
	Atp6v0a1	-0,52	2,15E-02	-0,21	4,10E-01
	Atp1b3	-1,10	9,66E-03	-0,48	8,69E-01
	Sec22b	-0,54	6,35E-02	0,78	2,19E-01
	Calu	-1,76	0,00E+00	-0,68	1,67E-03
	Mreg	N/A	7,85E-01	N/A	8,42E-01
	Ppib	-0,92	6,32E-03	-0,20	1,73E-01
	Pdia3	-0,41	1,01E-01	0,00	9,96E-01
	Slc3a2	-0,92	0,00E+00	-0,10	6,93E-01
	Trpv2	N/A	6,71E-01	0,00	1,00E+00
	Ywhab	-0,68	3,46E-02	-0,09	7,84E-01
	Ywhae	-0,55	3,02E-03	-0,03	8,79E-01
Not specifically deregulated	Atp6v1b2	-0,39	5,80E-02	-0,58	4,65E-03
	Rab27a	-1,00	2,31E-03	-1,03	2,51E-03
	Anxa2	-0,84	3,55E-02	-0,58	2,59E-03
	Gna13	-0,39	5,63E-02	-0,20	5,73E-01
	Dct	-1,68	0,00E+00	-1,55	0,00E+00
	Gpnmb	-1,48	3,00E-03	-0,78	1,98E-03
	Lamp1	-1,66	0,00E+00	-0,48	3,00E-02
	Myo5a	-1,12	0,00E+00	-0,54	6,62E-03
	Myo7a	-0,70	1,14E-02	-0,89	0,00E+00
	Ncstn	-0,62	6,42E-02	-1,22	0,00E+00
	Pdia4	-1,13	2,47E-02	-0,41	1,07E-02
	Pdia6	-0,34	1,21E-01	-0,49	7,40E-03
	Snd1	-0,28	1,23E-01	-0,45	2,72E-03
	Sytl2	-1,00	0,00E+00	-0,68	3,49E-03
	Tyr	N/A	8,57E-01	N/A	8,84E-01

Exosome-like BAG6KO-EVs

		EV proteome		Cellular proteome	
	**Gene names**	**log2 FC BAG6KO/WT**	**q-value**	**log2 FC BAG6KO/WT**	**q-value**
Specifically deregulated in EVs	Chmp2a	0,958249	2,07E-02	0,0050621	9,99E-01
	Chmp2b	1,17935	2,35E-03	N/A	8,25E-01
	Chmp4b	1,0868	2,12E-03	0,363785	9,07E-02
	Chmp5	1,33184	1,22E-02	-0,057766	8,78E-01
	Tsg101	1,03054	2,61E-03	-0,280121	1,01E-01
	Vps28	0,742254	1,10E-02	-0,159735	4,48E-01
	Vps36	0,910931	0,00E+00	-0,158687	4,01E-01
	Vps37b	1,21613	4,04E-03	-0,360706	2,71E-01
	Vps37c	0,784838	1,34E-02	-0,75221	4,49E-02
	Vps4b	0,812873	4,98E-03	-0,0346775	9,10E-01
	Vta1	0,755167	8,95E-03	0,466124	7,30E-02
	Vps4a	0,941038	1,28E-02	-1,07789	2,04E-03
	Ifitm3	2,56832	0,00E+00	0	1,00E+00
	Sh3gl1	0,573079	4,20E-02	-0,962532	0,00E+00
	Snf8	0,841376	9,95E-03	-0,10839	7,72E-01
	Sec31a	0,810083	0,00E+00	0,195366	1,66E-01
	Appl2	0,510428	6,90E-02	0,252258	1,45E-01
	Bst2	0,729434	3,89E-02	0	1,00E+00
	Grb2	0,575356	5,65E-02	0,415249	3,73E-02
	Mvb12a	0,92278	1,42E-03	-0,839358	2,54E-03
	Mvb12b	1,0013	4,11E-02	0	1,00E+00
	Sort1	0,948903	9,21E-03	-0,0265821	9,48E-01
	Eea1	1,32199	8,42E-03	0,419401	5,76E-02
Not specifically deregulated	Lrp1	0,952581	2,27E-03	1,40089	8,82E-04
	Arfgef2	0,426093	8,55E-02	-0,122036	3,22E-01
	Cd68	2,96219	5,34E-03	N/A	9,91E-01
	Ehd3	0,965844	2,19E-02	0,308753	9,69E-02
	Ecm29;AI314180	0,578709	9,99E-03	0,347452	5,71E-03
	Flot1	0,961083	1,31E-02	0,773437	1,08E-02
	Grb2	0,575356	5,65E-02	0,415249	3,73E-02
	Pacsin2	1,25372	1,39E-03	0,597606	6,54E-03
	Stx7	0,925775	1,09E-02	0,640253	4,73E-03
	Snx2	0,179757	1,74E-01	0,0277119	8,93E-01

Figure S2. Supplement related to Figure 2.

(A) Immunoblot analysis of CRISPR-generated BAG6KO B-16V cell clones (numbered 3, 5 and 8) probing for BAG6 and actin as a loading control. Immunoblot analysis for the loss of the BAG6 protein was performed in regular intervals during cell propagation and after thawing of cells.

(B) Sequencing analysis of three selected BAG6KO clones aligning the mutant sequence with the sequence obtained for the WT cell line. Sequencing was performed on a PCR product of 537 bp length cloned into the pUC18 plasmid and sequenced with both forward and reverse (M13-F20 and M13R) sequencing primer.

(C) Left graph: NTA analysis of 48h EV release from B-16V WT and BAG6KO cells under normoxic and hypoxic (1% O_2) conditions. Bar graphs represent mean ± SEM of three independent experiments. Right graph: Quantification of EV-associated BAG6 by ELISA (normalized to EV vesicle count) purified by ultracentrifugation at 100k x g from

B-16V cells that were either non-treated or cultured under hypoxic conditions (1% O_2). 2h EV release collections from B-16V WT cells after heat-shock for 40 min at 42° were used as a positive control and B-16V BAG6KO EVs served as a negative control. Bar graphs represent mean ±SEM of three independent experiments.

(D) RNAseq data-based waterfall plot showing the log2 fold change of up- and downregulated transcripts in EVs isolated from hypoxia-stressed BAG6KO B-16V cells compared to WT cells. RNAseq was performed using EVs of three independent EV purifications from each WT and BAG6KO B-16V cells.

(E) Overview of functional groups given as percentages of the total dataset based on significantly up- and downregulated RNAs.

(F) Plot showing the log2 change BAG6KO over WT proteins detected by mass spectrometry in EVs compared to their respective cells (whole cell lysate, WCL). Cut-off lines are indicated and proteins that are not significantly (FDR ≤ 0.5) deregulated in BAG6KO cells compared to WT cells, but which show at least 2-fold change difference in EV expression are highlighted in red.

(G) Western blotting of B-16V WT-EVs and BAG6KO-EVs (18 µg protein per lane) to validate differential expression of ANNEXIN A1, CHMP2B, VPS4B and VTA1 quantified by mass-spec analysis. Antibodies: ANNEXIN A1 (ab2114486, Abcam); CHMP2B (ab157208, Abcam); VPS4B (ab224736; Abcam); (VTA1 PA556605, Thermo Fischer Scientific).

(H) Lists of proteins detected by mass spectrometry clustering into melanosome-like (WT) EVs and exosome-like (BAG6KO) EVs and comparison to their respective cellular levels. WT, wild-typeM; kDa, kilodalton; NT, non-treated; n.d., not detected.

Other supplement files related to Figure 2:
Table S1. Transcriptomics of WT- and BAG6KO-EVs
Table S2. Proteomics of WT and BAG6KO B-16V EVs and cells

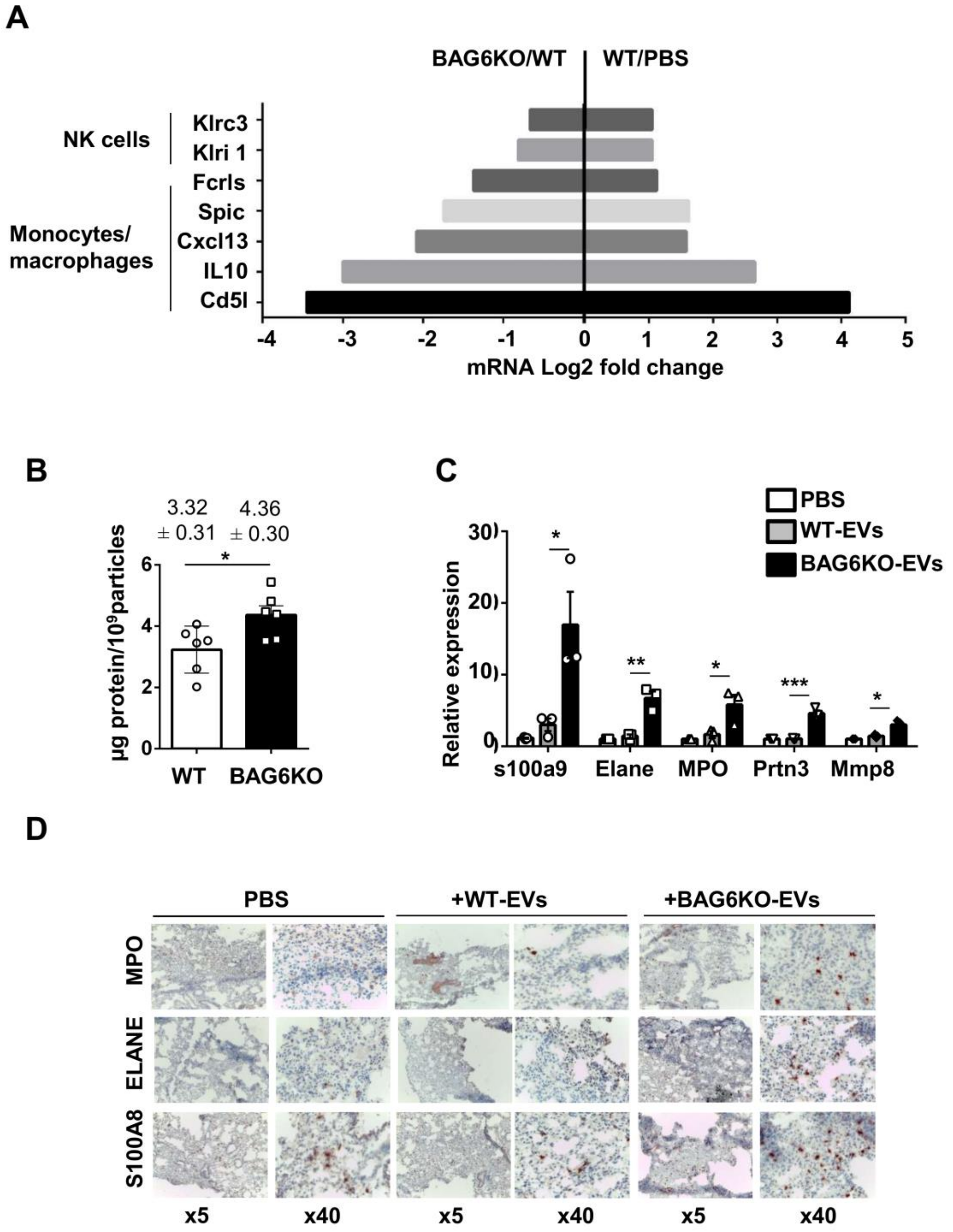
A
BAG6KO/WT
WT/PBS
NK cells
Klrc3
Klri 1
Fcrls
Spic
Monocytes/
macrophages
Cxcl13
IL10
Cd5l
-4
-3
-2
-1
0
1
2
3
4
5
mRNA Log2 fold change
B
3.32
± 0.31
4.36
± 0.30
*
µg protein/10⁹particles
6
4
2
0
WT
BAG6KO
C
PBS
WT-EVs
BAG6KO-EVs
Relative expression
30
20
10
0
*
**
*
***
*
s100a9
Elane
MPO
Prtn3
Mmp8
D
PBS
+WT-EVs
+BAG6KO-EVs
MPO
ELANE
S100A8
x5
x40
x5
x40
x5
x40

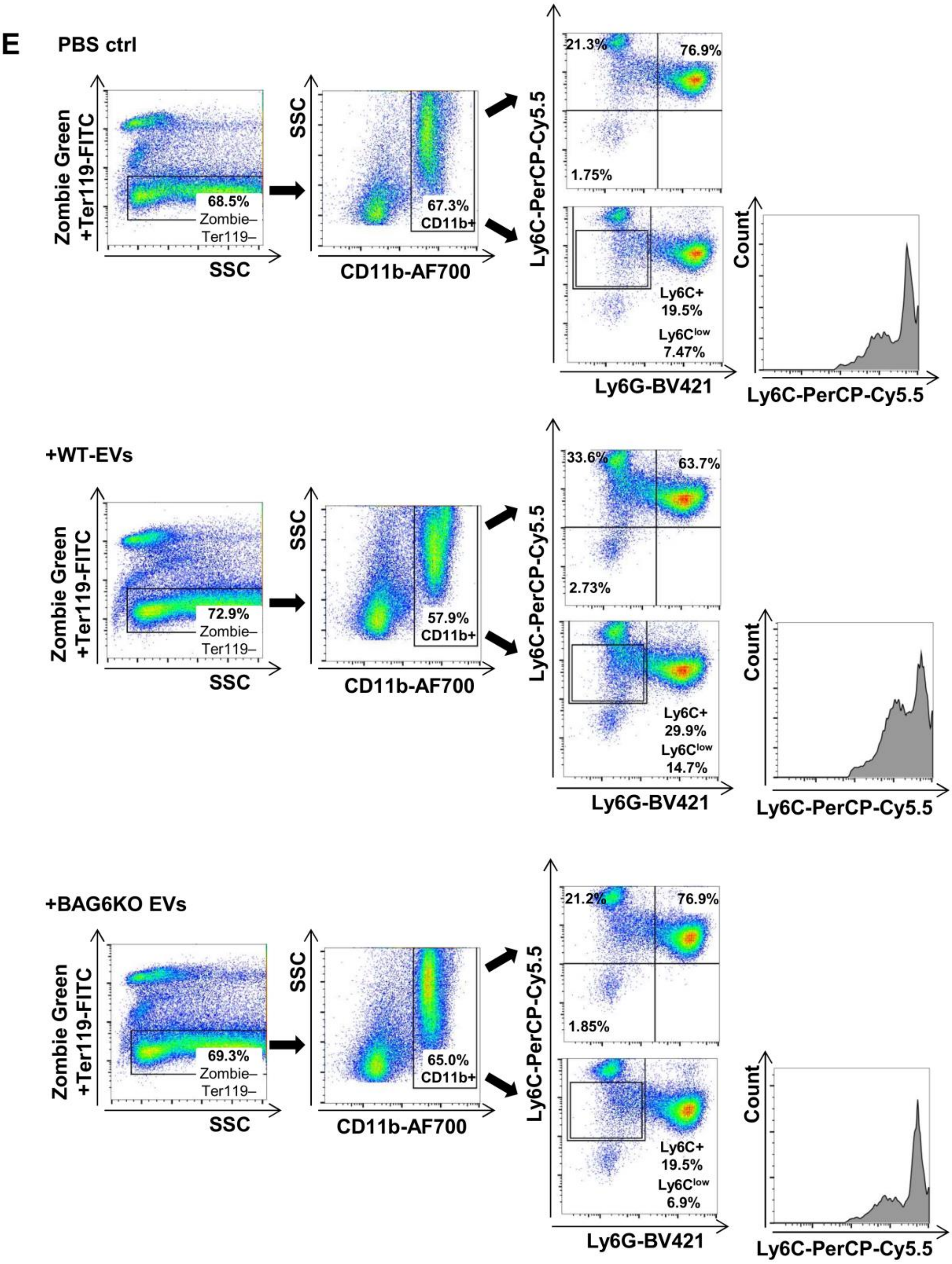
E
PBS ctrl
Zombie Green +Ter119-FITC
68.5%
Zombie–
Ter119–
SSC
67.3%
CD11b+
CD11b-AF700
Ly6C-PerCP-Cy5.5
21.3%
76.9%
1.75%
Ly6C+
19.5%
Ly6Clow
7.47%
Ly6G-BV421
Count
+WT-EVs
72.9%
57.9%
33.6%
63.7%
2.73%
29.9%
14.7%
+BAG6KO EVs
69.3%
65.0%
21.2%
76.9%
1.85%
19.5%
6.9%

F

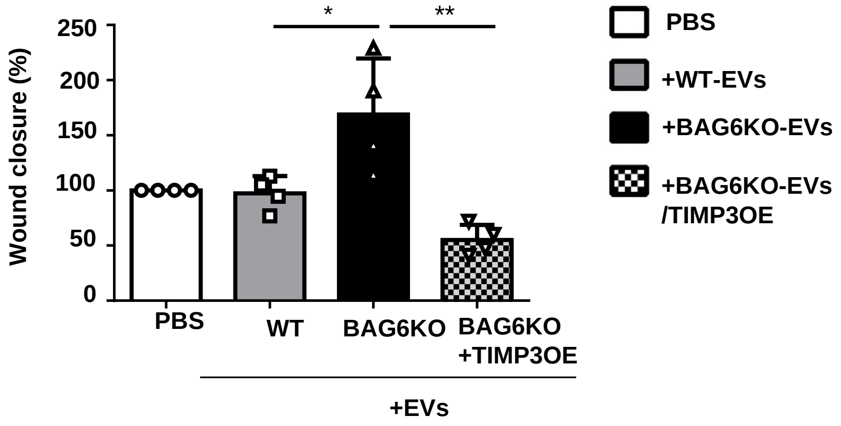

G

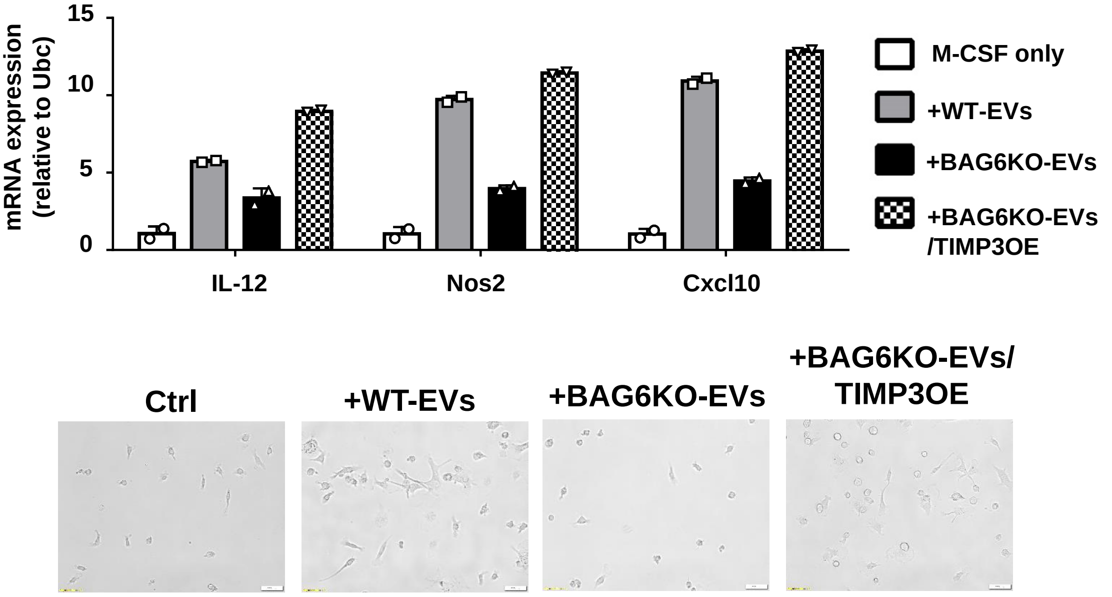

Figure S3. Supplement related to Figure 3.

(A) Bar graph representing the log2 fold change of mRNAs upregulated in lungs of mice treated with WT-EVs compared to both lungs of PBS control-treated mice (WT/PBS) and lungs of BAG6KO-EV-treated mice (BAG6KO/WT). The EV treatment plan of this experiment is provided in Figure 3A.

(B) Determination of protein content in WT- compared to BAG6KO-EVs isolated by ultracentrifugation and used for *in vivo* treatment experiments. Protein concentration was determined by nanodrop2000 and normalized to the particle concentration determined by NTA analysis. Graphs represent mean ± SEM of measuring 6 independent experimental samples.

(C) qRT-PCR analysis validating the indicated hits identified by RNAseq of lungs from mice treated according to the treatment plan provided in Figure 3A (n=3 mice per group). Bar graphs represent mean ± SEM.

(D) Immunohistochemistry using specific antibodies against S100a8, ELANE and MPO of lung tissue from mice that were treated with B-16V WT-EVs, BAG6KO-EVs or PBS according to the treatment plan presented in Figure 3A. Stainings represent the lung tissue of one out of three mice per group.

(E) Representative gating strategy of myeloid cells in the bone marrow of mice educated with EVs or PBS as a control. Dead cells and erythrocytes remaining after ACK lysis were excluded by staining with Zombie viability dye and Ter119 antibody, respectively.

CD11b, Ly6C and Ly6G staining was done to delineate macrophages (CD11b+Ly6C–Ly6G–), monocytes (CD11b+Ly6C+/lowLy6G–) and neutrophils (CD11b+Ly6C+Ly6G+).

(F) Wound healing scratch assay of 24h using B-16V cells incubated with EVs isolated from either WT B-16V cells, BAG6KO B-16V cells or BAG6KO B-16V cells transfected with TIMP3. PBS was used as a control. Bar graphs represent mean ±SEM of four independent experiments.

(G) qRT-PCR analysis of indicated M1 macrophage markers after 7 days of *in vitro* macrophage differentiation (+M-CSF) of mouse bone marrow-derived monocytes in the absence or presence of WT-EVs, BAG6KO-EVs or BAG6KO-EVs derived from TIMP3 transfected BAG6KO B-16V cells. Bar graphs represent mean ±SEM of two technical replicates and results are representative of three independent experiments. Representative microscopic images of macrophages after 7 days are shown.

EVs, extracellular vesicles; WT, wild-type; SSC, side scatter; OE, overexpression.

Other supplement files related to Figure 3:
Table S3A,B. Transcriptomics of lungs from mice treated with either WT-EVs, BAG6KO-EVs or PBS.

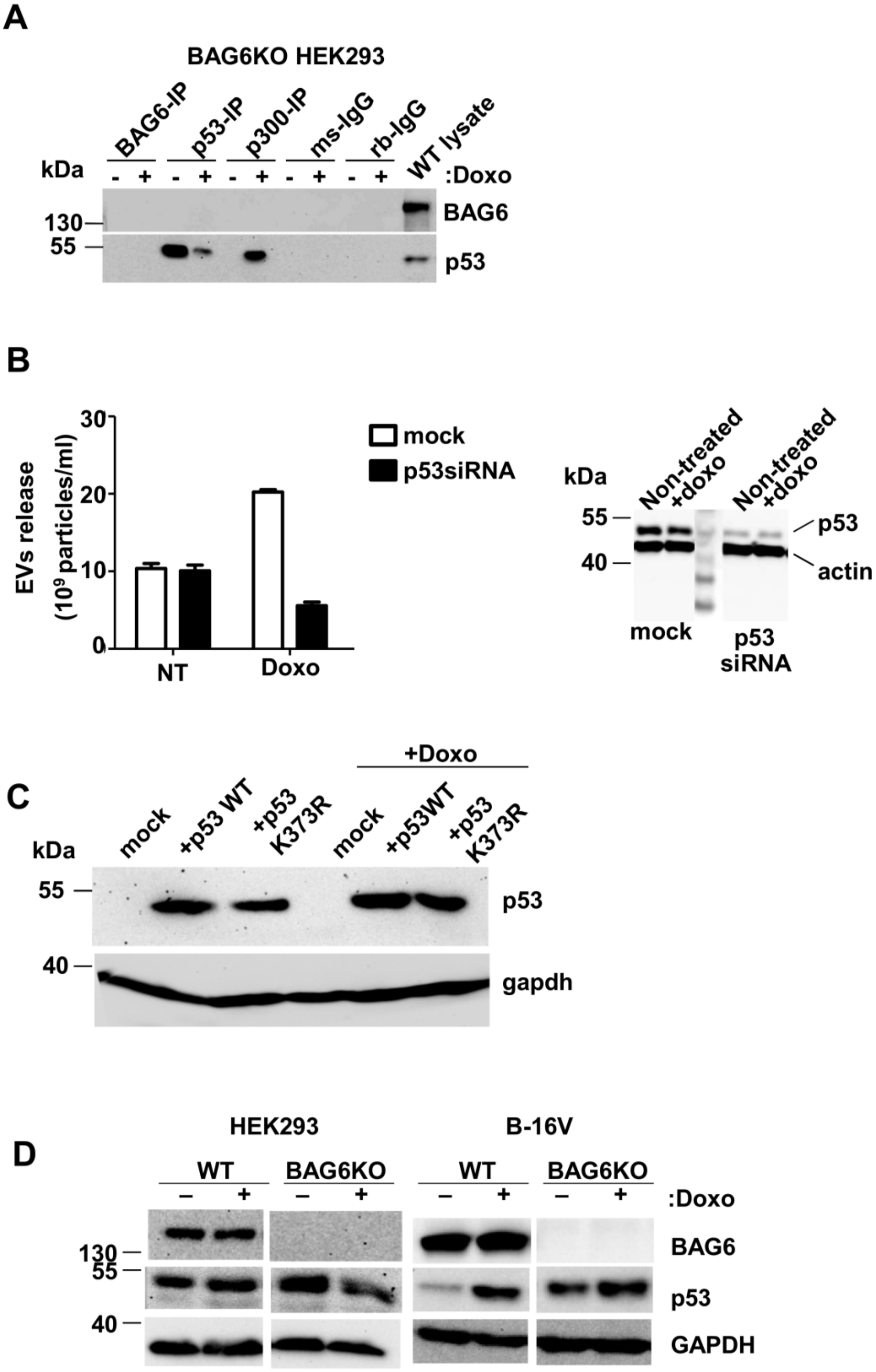

Figure S4. Supplement related to Figure 4.

(A) Immunoblot analysis of BAG6 and p53 in HEK293 BAG6KO cell lysates immunoprecipitated with anti-BAG6, anti-p53 or anti-p300 specific antibodies after treatment with 1 µM doxo for 1h or left untreated. Immunoprecipitation with either mouse (ms) or rabbit (rb) IgG isotype controls was performed as control and HEK293 WT cell lysate was loaded as a control for antibody staining of the membrane.

(B) Immunoblot analysis of p53 in p53KO- HCT116 cells either mock-transfected or re-transfected with p53 WT or p53 acetylation mutant (+p53K373R) after treatment with

100 nM doxo or left non-treated. The blot is representative of three independent experiments.

(C) Analysis of the EV release by NTA from WT or p53 siRNA knock down (kd) HEK293 cells that were either non-treated or treated with 100 nM doxorubicin or LBH for 16h. Immunoblot for p53 and actin as a loading control is shown.

(D) Immunoblot analysis of p53 in WT and BAG6KO HEK293 and B-16V cells either non-treated (-) or treated with the indicated concentrations of doxo (+) for 1h. One representative experiment out of three experiments is shown.

EVs, extracellular vesicles; WT, wild-type; kDa, kilodalton; doxo, doxorubicin; IP, immunoprecipitation; ms, mouse; rb, rabbit; IgG, immunoglobulin G.

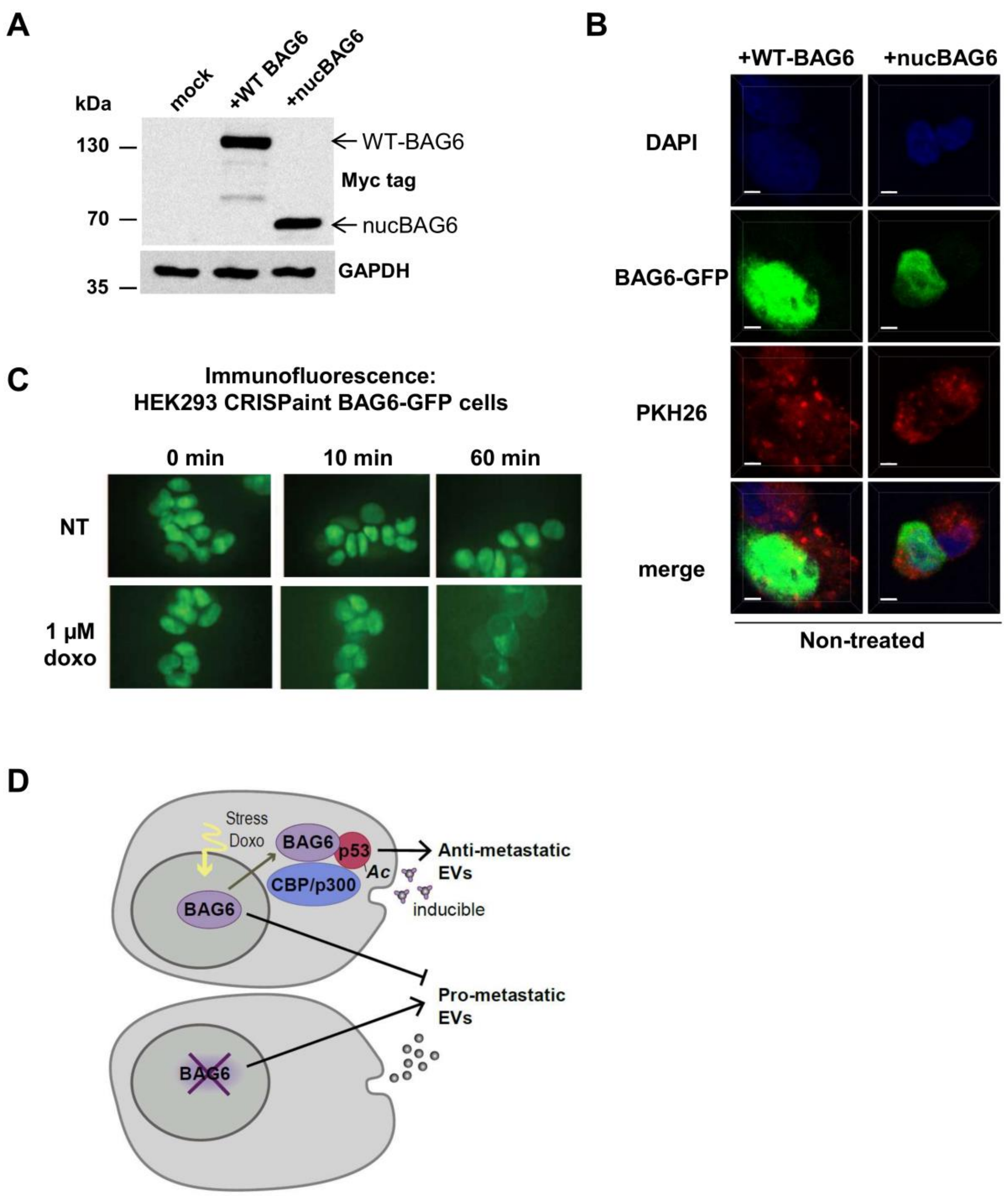

Figure S5. Supplement related to Figure 5.

(A) Immunoblot analysis of HEK293 BAG6KO cells transfected with full-length BAG6 (+WT BAG6) or with a N-terminal deleted BAG6 mutant (+nucBAG6), detected by using a myc-tag specific antibody. Probing for GAPDH was done as a loading control. The blot represents one out of three independent experiments.

(B) Immunofluorescence microscopic analysis of HEK293 cells transfected with either full length BAG6 (+WT BAG6) or N-terminal deleted BAG6 mutant (+nucBAG6). Transfected BAG6 was visualized by a GFP-tag, the nucleus and cell membrane were visualized by staining with DAPI and PKH, respectively, and merged images are shown. Scale bar: 2 µm.

(C) The CRISPaint method was used to fuse a GFP-tag to the endogeneous BAG6 gene (C-terminus) and the cellular localization of the BAG6-GFP protein was monitored in living cells using a inverse spinning disc microscope (Zeiss) with 5% $C0_2$/37°C. A 8-chamber slide and a 40x objective with a NA of 1.1 (0.333µm/pixel) were applied. Cells were either left non-treated or incubated with 1µM doxo and pictures were taken at the indicated time points.

(D) Schematic illustrating the impact of BAG6 expression and subcellular localization on the EV release.

WT, wild-type; kDa, kilodalton; DAPI, 4′,6-Diamidin-2-phenylindol; GFP, green fluorescent protein.

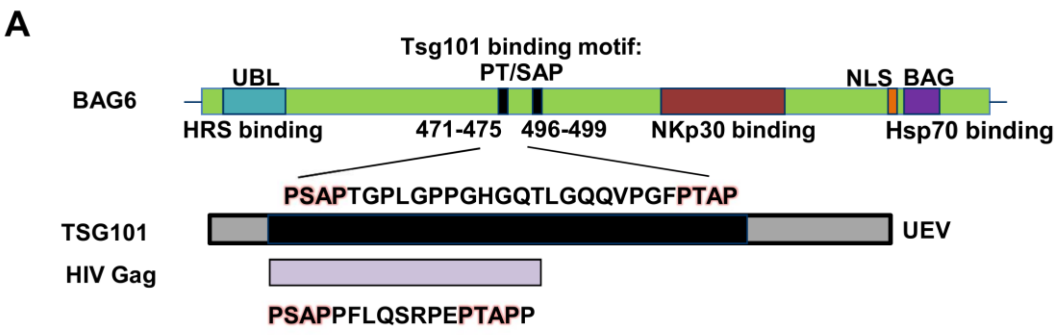

Figure S6. Supplement related to Figure 6.

(A) Schematic representation of BAG6 protein domains highlighting its PT/SAP motif in analogy to the corresponding element found in HIV gag binding to the TSG101 UEV domain.UBL, ubiquitin-like domain; NLS, nuclear localization signal; BAG, Bcl-2-associated athanogene domain; UEV, Ubiquitin E2 variant domain; HRS, hepatocyte growth factor-regulated tyrosine kinase substrate; NKp30, Natural cytotoxicity receptor 30; Hsp70, Heat shock protein 70.

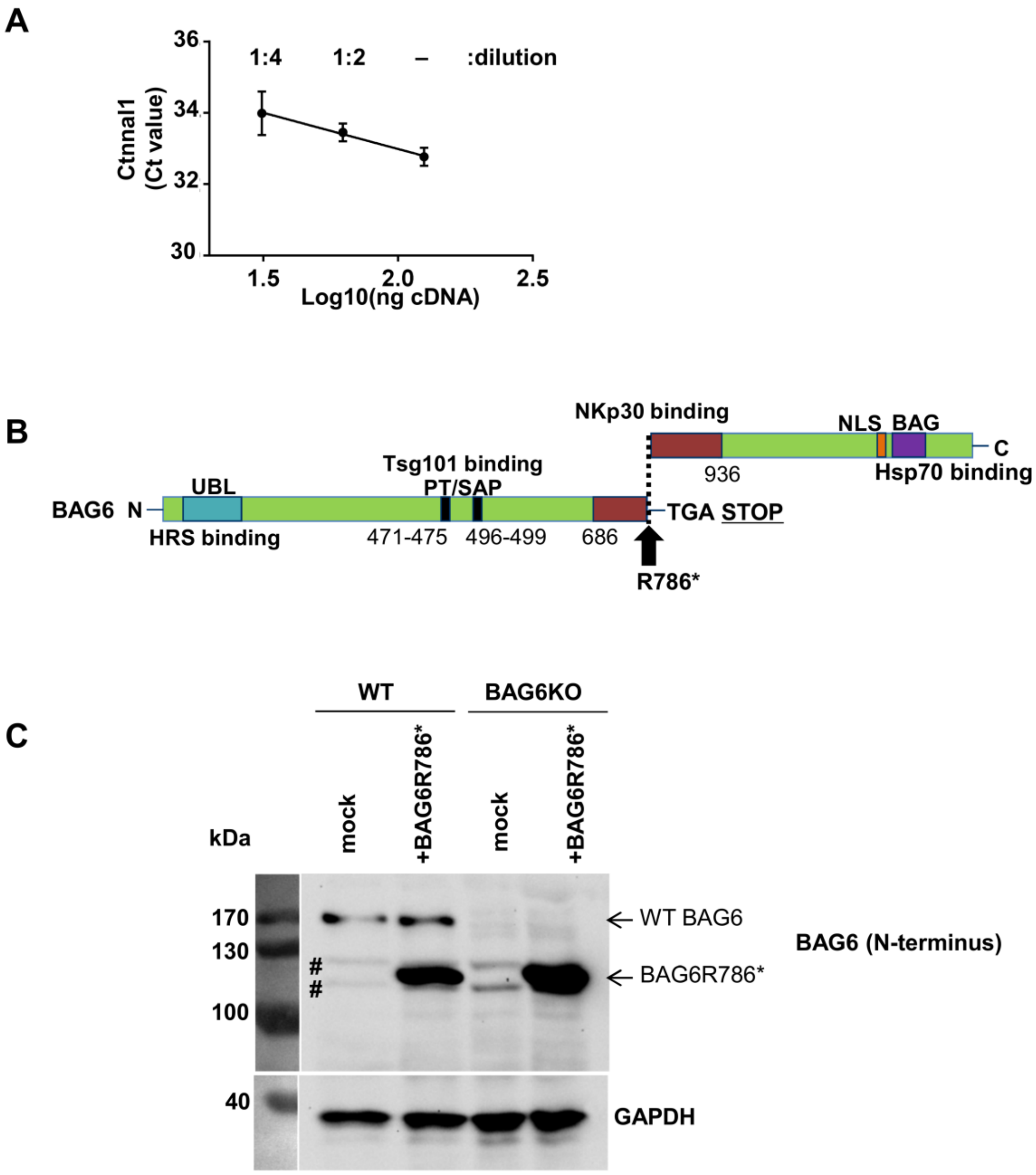

Figure S7. Supplement related to Figure 7.

(A) Test of qRT-PCR assay robustness using cDNA made from RNA isolated out of melanoma patient plasma EVs. The graph depicts a dilution series of EV cDNA using RNA isolated from three plasma samples (mean ± SEM) and experiments in Figure 7B were performed at equal concentrations, either undiluted or diluted 1:2.

(B) Schematic representation of the BAG6R786* mutant protein.

(C) Immunoblot analysis of BAG6R786* mutant protein transfected into WT or BAG6KO HEK293 cells using a BAG6 N-terminus-specific antibody which also detects the endogenuous full length BAG6 protein (WT BAG6). GAPDH was probed for as a loading control. # indicates weak non-specific bands. The blot is representative for two independent experiments.

EVs, extracellular vesicles; SEM, Standard error of the mean; UBL, ubiquitin-like domain; NLS, nuclear localization signal; BAG, Bcl-2-associated athanogene domain; HRS, hepatocyte growth factor-regulated tyrosine kinase substrate; NKp30, Natural cytotoxicity receptor 30; Hsp70, Heat shock protein 70; WT, wild-type; kDa, kilodalton; BAG6R786, BAG6 mutant with stop codon at Arginin position 786.

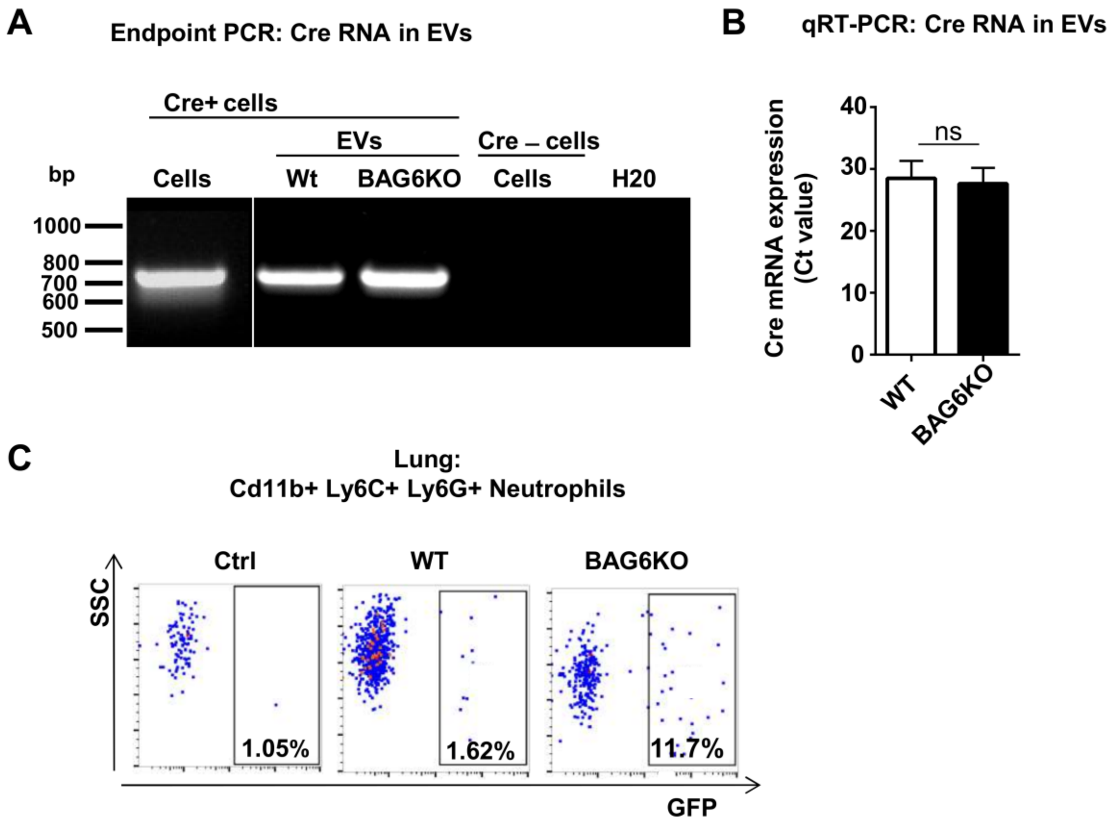

Figure S8. Supplement related to the discussion.

(A) Cre mRNA detection by PCR of EVs isolated from WT and BAG6KO cells stably transfected with pcDNA3.1 CMV-CFP-Ubc-Cre-zipcode-zeo plasmid (kindly provided by Jacco van Rheenen, Utrecht, Netherlands). cDNA from cellular RNA and cDNA of EVs from Cre-negative cells was used as a positive and negative control, respectively. H20 served as a technical negative control.

(B) qRT-PCR analysis comparing the Cre mRNA levels detected in WT versus BAG6KO B-16V EVs. Bar graphs represent the mean ± SEM of Ct values obtained using three independent EV samples. Equal amounts of cDNA were used in each run.

(C) Flow cytometric analysis of GFP converted Cd11b+ Ly6C+ Ly6G+ neutrophils by flow cytometry of cells isolated from the lung obtained from tumour-burden mTmG reporter mice i.v. transplanted with WT or BAG6KO Cre-expressing B-16V cells or treated with PBS.

Figure S9. Summary model
See discussion for explanation

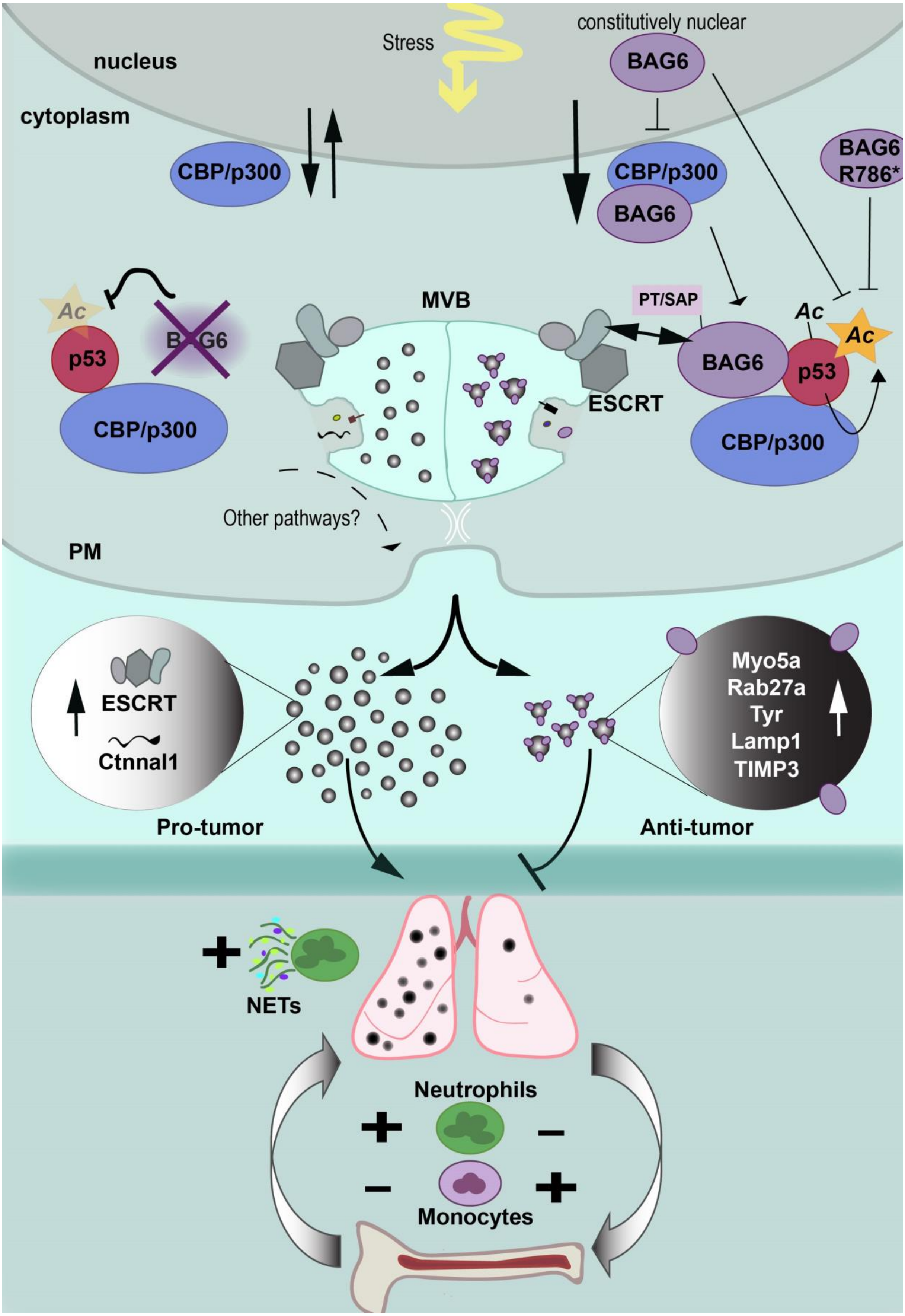

Extended Materials and Methods

Cell culture and culture conditions

HEK293 (ACC-305) and B-16V (ACC-370) cell lines were maintained according to the DSMZ guidelines in DMEM and RPMI medium, respectively. HCT116 cell lines, kindly provided by Annette Paschen, Essen, Germany, were maintained in McCoy medium. All media were supplemented with 0.5% Penicillin-Streptomycin and 10% fetal bovine serum. Cell lines were kept under standard culturing conditions with 5% CO_2, 37°C or under hypoxic conditions at 1% oxygen saturation and 5% CO_2, 37°C. Cell lines were regularly tested for mycoplasma contamination.

Mice

C57BL/6J mice were purchased from Charles River and all experiments were approved by the state authorities of North Rhine-Westphalia (number 84-02.04.2013.A.073). For EV injections (Figure 3A-B), two male and one female mice between 8-12 weeks of age were used. For EV education experiments (Figure 3C-E), experiments were performed with female mice starting at an age of 10 weeks.

p53-proficient and deficient murine CLL models were derived from the established Eμ:Tcl1a-driven CLL model [1]. In brief, Eμ:Tcl1a mice were crossed with Cd19:Cre [2] and Tp53fl/wt mice [3] to generate Eμ:Tcl1a; Cd19:Cre; Tp53fl/fl-compound mutant mice [4].

Human samples

Plasma were obtained with informed written consent by the patients and approval by the local ethics committee of the University Hospital Essen [ref. no. 11-4715].

CRISPR/CAS9-mediated knockout cell line generation

Generation of HEK293 BAG6KO and CBP/p300 double KO were described [5]. Knockout of BAG6 in B-16V cells was performed using the Zhang Lab's reagents [6, 7] according to their protocol. Briefly, target sequences for sgRNA were designed using the Zhang Lab design tool (available at http://crispr.mit.edu/). Cells were transfected with px330 (nuclease approach) expression plasmid (Addgene #42230) and screened for protein loss by Immunoblotting. In experiments using WT or BAG6KO B-16V cells, three BAG6KO clones were pooled and compared to three isolated WT clones.

In vivo treatment experiments and sample preparation from experimental mice

8-12 weeks old C57BL/6J mice, housed and fed under pathogen-free conditions, were injected with EVs or cells intravenously into the tail vein using 27 gauge (EVs) or 26 gauge (cells) needles according to the time treatment schedule provided in Figure 2A and 2D. Blood sampling was done by tail vein scratching. For flow cytometric analysis of cells from EV-treated and/or B-16V metastasis-bearing C57BL/6J mice, single cell suspensions of spleen, blood and bone marrow were generated. Cut spleen pieces and bone marrow-flushed cells were passed through a 100 µm mesh and single cell suspensions as well as blood were incubated in erythrocyte lysing buffer (ACK) for 5 minutes and washed in PBS before antibody staining for flow cytometry. Dissected mouse lungs were either frozen in optimal-cutting-temperature compound (OCT Tissue Tec) on dry ice and stored at -80°C for immunohistochemistry or directly taken up in RNA stabilization solution and frozen at -20°C until further processed. For RNA isolation, lung tissue was homogenized using the gentleMACS™ dissociator according to the manufacturer's instructions (M-tubes, MACS Program RNA_02) and the RNeasy mini kit according to the manufacturer's instructions. On-column DNase digestion was performed.

Murine splenocytes from Eμ:Tcl1a;Cd19:Cre mice were purified and cultured as described [8]. The minimum purity of CD5+CD19+ B cells from mice was 85%. Purified splenocytes were cultured for 24 h in Panserin 411S media (Pan-Biotech, Germany) and treated with 100 nM doxorubicin for 16 h. Supernatants were collected and exosome purification was performed by sequential ultracentrifugation. Viability of cells was confirmed (>90%).

Cell treatment

Cell lines were treated for 16 h with 100 nM doxorubicin or 100nM LBH, respectively. For short term (5– 120 min) DNA damage induction, cells were treated with 1 or 10 µM doxorubicin (as stated in the figure legends).

Plasmids and transfection

pcDNA3.1 wild-type BAG6 (BAT3) and nucBAG6 are described [9]. pcDNA3.1 BAG6R786* was generated by cloning the stop codon TGA at position 2337 of the full-length BAG6 sequence introduced by site-directed mutagenesis using QuickChange II Site-Directed Mutagenesis Kit and the expression plasmid pcDNA3.1 wild-type BAG6 as a template. HRS expression vector pCS2 was obtained from Addgene (#29685) and pcDNA3.1 for p53 was kindly provided by Dr. Pattingre (INSERM, France). Transfection was performed using Lipofectamine 2000 or jetprime according to the manufacturer's instructions.

Antibodies

All antibodies used are listed in Supplementary Table S4.

Flow cytometry

Fluorescence-activated cell sorter (FACS) was performed on a FACSCalibur or Gallios. Cells were stained with either directly-labelled antibodies, PE- or DyLight649-labelled goat anti-mouse or donkey anti-rabbit secondary antibodies as indicated in the figure legends. Flow cytometry was used to assess cell death (7AAD/Annexin V staining), EV expression levels of CD9, CD81, CD82, CD63, BAG6, phosphatidylserine, and acetylation of H3K18, H3K9 or p53K373 using specific antibodies. EVs were bound to polystyrene beads prior to flow cytometry as previously described [10]. Flow cytometric analysis of intracellular proteins was performed after fixation of cells with 4% formaldehyde in PBS for 12 minutes at 37°C, cooled down on ice for 1 minute and subsequently permeabilised with methanol for 30 minutes on ice prior to antibody staining. Single cell suspensions of mouse spleen, bone marrow and blood cells were stained with Zombie Aqua^{TM} and Ter119 to exclude dead cells and remaining erythrocytes, respectively, and specific antibodies against CD11b, Ly6C and Ly6G were used to differentiate between the different myeloid cell populations. A minimum of 1,000 events was counted in FACS measurements. FACS data were analyzed with FlowJo software.

***In vitro* expression assay**

In vitro expression experiments were performed using cell-free protein expression kit based on Leishmania tarentolae. Recombinant proteins were obtained by cloning in pLEXSY-invitro vector.

Immunoprecipitation

Cell lysates or *in vitro* expressed proteins were precipitated using specific antibodies against BAG6, p53, CBP/p300, HRS, ubiquitin or acetyl-lysine. A minimum of 1 µg of antibody were used for 100 µg of total protein and Protein A magnetic beads were used for pull-down.

Yeast Two Hybrid analysis

To confirm BAG6 interaction with the ESCRT protein TSG101, we used Gold Yeast two hybrid system. BAG6 and TSG101 were cloned into pGBT9 and pGADT7 expression vectors. Transformation, mating and binding analysis was done according to the manufacturer's instructions.

Microscopy

Immunofluorescence

HEK293 cells were transfected with pcDNA3.1 FL-BAG6 or CT-BAG6 using lipofectamine according to the manufacturer's instructions. 24h later cells were stained with PKH26 for membrane labelling and seeded onto glass slides. 3h later, cells were either left untreated or treated with 1 µM Doxo for 1h before fixation using 70% MeOH and blocking using PBS/10% FBS. The transfected BAG6 was detected by staining with an anti-myc antibody and subsequent visualisation by anti-mouse-FITC. Images were acquired with a Leica DMi8 inverse microscope (Leica TSC SP8).

Immunohistochemistry

Frozen tissue blocks containing mouse lungs were sectioned in slices of 7 µm and slices were stained with DAPI to visualize cell nuclei and with specific antibodies against MPO, ELANE and S100a8 visualized by incubation with biotinylated secondary antibody and subsequent HRP-labelled streptavidin using KPL Histomark® Biotin Streptavin-HRP Systems Kit and ACE (red) substrate kit according to the manufacturer's instructions. Sections were finally stained with hematoxylin before embedment with Aquatex. Images were aqcuired with a Leica Type DM1000 LED.

Microscopy images were analysed with Leica Application Suite X 3.1.5.16308 and Imaris 8.3.1 software (3D reconstruction and cropping).

ELISA

The mouse monoclonal 3E4 (raised against the BAG6 *N*-terminus) and the BAG6-specific chicken polyclonal 13pp2 antibodies (own lab) were used to detect BAG6 in a sandwich ELISA procedure as previously described(10).

EV preparation

EVs were collected in cell culture for either 24 or 48h in either EV-depleted medium (overnight centrifugation at 100,000 x g) or protein-free CD293 medium. For quantification, EVs were purified by sequential centrifugation. The centrifugation protocol included consecutive pre-centrifugation steps at 300 x g (10 min), 2,000 x g (10 min) and 3,500 x g (20 min) for clearance of cells and cellular debris before ultracentrifugation at 10,000 x g (60 min) and/or 100,000 x g (90 min) using SW 41 Ti rotor or Type 45Ti) for at least two times with intermediate resuspension in PBS or HBSS and ultra-centrifugation at the respective g force using TLA-45 rotor in the last centrifugation. EVs were resuspended in PBS or HBSS. The amount of EV protein was quantified by Nanodrop 1000 and/or using BCA assay. The number of particles was determined by Nanoparticle Tracking Analysis. In experiments analyzing EV release, we have seeded equal numbers of cells and the measure of EVs released per cell is based on the end of the conditioning period to also account for cell growth during this period. RNA from EVs was isolated using the RNeasy Mini Kit according to the manufacturer's protocol recommended for processing animal cell lysates. The viability of EV-releasing cells was regularly checked by both trypan blue staining and microscopic analysis as well as by 7AAD/Annexin5 staining and flow cytometry.

Free flow electrophoresis (FFE)

Conditioned CD293 medium from either BAG6-transfected or non-transfected HEK293 cells was sent to FFE Service GmbH (Feldkirchen, Germany) for analysis on a FFE NextGen to fractionate vesicles from soluble proteins. Samples were thawn and 100 ml were concentrated to 4-7 ml by tangential flow ultrafiltration using a Microkros hollow fiber module (Spectrum Lab, C02-E300-05-N) at room temperature. Concentrates were then subjected to continuous Zone Electrophoresis (ZE) with 3 min separation time at 1000 V in a horizontal chamber position (500 mm x 100 mm, 0.2 mm gap). Fractions of typically 200 µL were collected in black flat bottom 96-well plates (Greiner microloan Fluotrac 200) or protein low bind polypropylene deep well plates with 2 mL per well and read in a microplate reader (Tecan M200) equipped for UV-VIS and fluorescence spectroscopy. A mixture of dyes of different isoelectric points (pI) in the range of pI 4-10 was used in system suitability testing and read at 420 nm, 517 nm and 595 nm. Proteins were detected by top count fluorescence at 280 nm (excitation 350 nm, emission 10 nm bandwidth) and a photomultiplier gain setting of 80. pH measurements on microplate were performed with a robotic system equipped with a pH-meter and electrode. Fractions obtained by FFE were analysed for protein concentrations BCA assay and for particle concentration by NTA. Particle-containing fractions were analysed by bead-assisted flow cytometry for the presence of BAG6 and the vesicle markers CD81 and HSP70.

Quantitative RT-PCR

RNA from EVs or lung tissue was reverse transcribed with RevertAid First Strand cDNA Sysnthesis Kit using oligo-d(T) primers and/or random primers. Quantitative PCR measurements were performed on a 7500 real-time PCR system using SYBR Green. Initial heat inactivation was 95°C for 15 min and 40 cycles of 15 sec at 94°C, 30 sec at 56°C, and 30 sec at 72°C were performed followed by melting curve analysis. All primers used are listed in supplement Table S5.

Western Blot

Cells or EVs were lysed in buffer (50 mM Tris-HCl pH 8, 150 mM NaCl, 0.5% Triton X-100, 0.5% protease inhibitors). Cytoplasmic extraction of lysates was performed using soft lysis conditions for 15 minutes with Triton X-100 lysis buffer followed by sequential centrifugation. Protein concentration was measured with a BCA protein assay. 10 to 30 µg were loaded onto sodium dodecyl sulfate-polyacrylamide gel electrophoresis gels for blotting using standard methods. Blots were incubated with primary antibody BAG6, p53, ALIX, CD81, HRS, flotillin-1, and p300 and TSG101.

The western blots were developed using X-ray films or by detection with a CCD camera. For quantification, rolling-ball background subtraction was applied and band intensity was quantified using GelQuantNET.

Scratch assay

B-16V WT cells were seeded onto 24-well plates and scratch wounds were made using a 200 µl pipette tip 24h post seeding when cells reached around 80% confluency. After wounding, cells were washed two times with PBS and cells were incubated in RPMI and 10 µg of EVs from B-16V WT cells, BAG6KO cells or BAG6KO cells transiently transfected with Timp-3 plasmid using lipofectamine or PBS as a control. Microscopic pictures were taken at 5x magnification of at least 3 different spots along the scratch immediately after wounding and 24h later. The experiments were performed in 3 technical replicates and repeated 4 times in independent biological replicates.

Timp-3 plasmid: Mouse TIMP-3/TIMP3 Gene ORF cDNA clone expression plasmid, C-His tag, Biozol (article number SIN-MG57384-CH-1)

Macrophage differentiation and polarization

Bone marrow cells were seeded in 12 well plates at 10^6 cells/well and monocytes were allowed to adhere in the presence of 20 ng/ml M-CSF in RPMI medium. After 24h, cells in suspension were removed and adherent cells were washed thourougly before cells were incubated in RPMI medium supplemented with M-CSF (control) or additionally with 10 µg/ml EVs isolated from either WT, BAG6KO or BAG6KO cells transiently transfected with Timp-3 plasmid using lipofectamine. Medium was refreshed every second day until day 7 where cells were lysed in the plate and processed for RNA isolation. qRT-PCR was performed using primers against macrophage M1 markers IL-12 (IL-12 for ACGCAGCACTTCAGAATCAC and IL-12 rev CGCAGAGTCTCGCCATTATG); Nos2 (Nos2 for GGCAGCCTGTGAGACCTTT and Nos2 rev TTGGAAGTGAAGCGTTTCG); and Cxcl-10 (Cxcl-10 for GACGGTCCGCTGCAACTG and Cxcl-10 rev CTTCCCTATGGCCCTCATTCT).

Three independent experiments were performed.

Mass Spectrometry

Samples were obtained by ultracentrifugation of CD293 supernatants of B-16V WT or BAG6KO cells or by harvesting the respective cells cultured under hypoxic conditions for 48h. 10 µg EV protein or cell lysate were lysed in 6M Urea/ 2M thiourea in Hepes buffer additionally aided by freezing at -80°C and thawing. Proteins were then linearised using 1:10 volume of 100 mM DTT for 30 minutes at RT and alkylated using 1:10 volume of 550 mM for 30 min and RT. Proteins were Lys-C digested for 2h at RT and afterwards solution was diluted to 1M urea using 50 mM ABC buffer for trypsin digestion overnight at RT (both digests at 1 to 100 enzyme to protein ratio). The reaction was stopped by acidification using 5% concentrated nitric acid/0.2% trifluoroacetic acid before peptides were desalted on a C18 Stage tip.

All samples were analyzed at the CECAD proteomics facility (Cologne, Germany) on a Q-Exactive Plus (Thermo Scientific) mass spectrometer that was coupled to an EASY nLC 1000 UPLC (Thermo Scientific). Briefly, peptides were loaded with solvent A (0.1% formic acid in water) onto an in-house packed analytical column (50 cm × 75 µm I.D., filled with 2.7 µm Poroshell EC120 C18, Agilent). Peptides were chromatographically separated at a constant flow rate of 250 nL/min using linear gradient (solvent B 0.1% formice acid in 80% acetonitrile) over 150 min gradients.

Mass spectrometry bioinformatics and statistical analysis

All mass spectrometric raw data were processed with Maxquant using default parameters. Briefly, MS2 spectra were searched against the Uniprot MOUSE database, including a list of common contaminants. False discovery rates on protein and PSM level were estimated by the target-decoy approach to 1% (Protein FDR) and 1% (PSM FDR), respectively. The minimal peptide length was set to 7 amino acids and carbamidomethyolation at cysteine residues was considered as a fixed modification. Oxidation (M) was included as variable modification. The match-between runs option was enabled. LFQ quantification was enabled using default settings. Downstream data processing was conducted within the Perseus computational platform. Briefly, protein groups flagged as „reverse“, „potential contaminant“ or „only identified by site“ were removed from the data. LFQ data were log2 transformed. Statistical analysis of differentially regulated proteins was performed using a two-sided t-test (fudge factor s0 was adjusted to 0.1). Resulting p values were corrected for multiple testing using a permutation-based FDR approach.

RNAseq

RNA quality was assessed using the Experion RNA StdSens Analysis Kit. RNA-seq libraries were prepared from total RNA using the TruSeq Stranded mRNA LT kit according to the manufacturer's instructions. Quality of sequencing libraries was controlled on a Bioanalyzer 2100 using the Agilent High Sensitivity DNA Kit. Pooled sequencing libraries were quantified with digital PCR (QuantStudio 3D) and sequenced on the HiSeq 1500 Illumina platform in Rapid-Run mode with 50 base single reads. RNAseq was performed from RNA isolated using the RNAeasy mini kit with the Illumina Truseq mRNA kit v2 on an Illumina Hiseq 1500 according to the manufacturer's instructions.

RNA-Seq Bioinformatic Analysis

Raw transcriptome reads were aligned to the mouse genome from Ensembl 89 [11] using STAR version 2.4.1a [12]. Gene expression was quantified on gene models that included only protein coding transcripts for protein coding genes using custom python scripts. Differential genes were called using edgeR [13] at a threshold of FDR <= 0.05; |log2FC| >= 1 and counts per million >= 3.

Statistical analysis

Statistical analyses were performed using Graphpad Prism software. Data are presented as mean ± SEM values of at least three biologically independent experiments or one representative experiment as indicated. *, ** and *** indicates P < 0.05, P< 0.001 and P<0.001. Two-tailed, unpaired Student's t-tests were performed to analyze the significance of mean values between two variables, if not otherwise stated. Statistical analysis of EVs education animal experiments was performed using non-parametric Kruskal-Wallis test (mean ranks compared with WT-EVs group) and Dunn's multiple comparisons test. Data are shown as mean ± SEM (n ≥ 8). An unpaired Welch's t-test was performed to analyze TCGA data to account for unequal sample sizes.

Data deposition

The mass spectrometry proteomics data have been deposited to the ProteomeXchange Consortium via the PRIDE partner repository with the dataset identifier PXD010677. RNAseq data of mouse lungs were deposited at ArrayExpress accession E-MTAB-7119. RNAseq data of B-16V EVs were deposited at ArrayExpress accession E-MTAB-7119.

Table S4. List of Resources

Reagent or Resource	Source	Identifier
**Antibodies**		
BAG6 (monoclonal mouse, 3E4; raised against N-term)	Own lab	Pogge von Strandmann, Simhadri et al., 2007
BAG6 (polyclonal chicken, 13pp2; raised against recombinant N-term)	Own lab	
Alexa Fluor700 rat anti-Cd11b (M1/70)	Biolegend, San Diego, USA	Cat# 101222
PerCP-Cy5.5 anti-Ly6C (HK1.4)	Biolegend, San Diego, USA	Cat# 128011
Brilliant violet 421 rat anti-Ly6G (1A8)	Biolegend, San Diego, USA	Cat# 127627
Fitc rat anti-Ter119 (Ly-76)	Biolegend, San Diego, USA	Cat# 116205

Rabbit anti-p53	Cell signaling, Danvers, Massachusetts, USA	Cat# 9282S
Rabbit anti-p53K373 (EP356(2)AY)	Abcam, Cambridge, UK	Cat#62376
Rabbit anti-p300 (C-20)	SantaCruz Biotech	Cat#sc-585
Mouse anti-beta actin (AC-15)	Sigma, Kawasaki, Japan	Cat# A5441
Rabbit anti-gapdh HRP (D16H11)	Cell signaling, Danvers, Massachusetts, USA	Cat# 8884
Rabbit anti-HRS	Bethyl Lab Inc, Montgomery, Texas, USA	Cat#A300-989A
Rabbit anti-TSG101	Abcam, Cambridge, UK	Cat# ab30871
Mouse anti-ALIX (3A9)	Biolegend**,** San Diego, USA	Cat# 634501
Mouse anti-flotillin-1 (RUO)	BD Biosciences, Franklin Lakes, New Jersey, USA	Cat# 610821
Mouse anti-human CD81 (TAPA-1)	Biolegend**,** San Diego, USA	Cat# 349501
Mouse anti-CD63	Biolegend**,** San Diego, USA	Cat# 353014
PE-anti mouse CD63 (NVG-2)	Biolegend**,** San Diego, USA	Cat# 143903
Mouse anti-CD9 (HI9a)	Biolegend**,** San Diego, USA	Cat# 312102
Alexa Fluor647 anti-mouse CD9	Biolegend**,** San Diego, USA	Cat# 124809
Annexin V – PE	Biolegend**,** San Diego, USA	Cat# 640908
Mouse anti-HSP70	SantaCruz Biotech., TX, USA	Cat# sc-66048
Mouse anti-Adam10 (EPR5622)	Abcam, Cambridge, UK	Cat# ab124695
Anti-myc tag	Biolegend**,** San Diego, USA	Cat# 626802
Goat anti-MPO	R&D systems, Minneapolis, Minnesota, USA	Cat# AF3667-SP
Rabbit anti- S100a8	Sigma, Kawasaki, Japan	Cat# HPA024372
Rabbit anti-Elane	Sigma, Kawasaki, Japan	Cat# HPA066836
PE-anti mouse IgG1	Biolegend**,** San Diego, USA	Cat# 406607
LEAF™ Purified Mouse IgG1, κ Isotype Ctrl	Biolegend**,** San Diego, USA	Cat# 400153
FITC Goat anti-mouse IgG (minimal x-reactivity) Antibody	Biolegend**,** San Diego, USA	Cat# 405305
Dylight^{TM} 649 donkey anti-rabbit IgG	Biolegend, San Diego, USA	Cat#405312
**Biological samples**		
Plasma from melanoma patients	Annette Paschen, Essen	N/A
**Chemicals**		
Doxorubicin	Sigma, Kawasaki, Japan	Cat# D2975000
Panobinostat (LBH589)	SantaCruz Biotech, Dallas, Texas, USA	Cat# sc-208148
Aquatex	Merck, Darmstadt, Germany	Cat#1085620050
**Medium and Solutions**		
RPMI	Life technologies (Gibco), Carlsbad, California, USA	Cat# 11875093
DMEM	Life technologies (Gibco), Carlsbad, California, USA	Cat# 10567014

McCoy’s	Life technologies (Gibco), Carlsbad, California, USA	Cat# 16600082
Panserin 411S	Pan-Biotech, Aidenbach, Bayer, Germany	Cat# P04-71411S
CD293	Life technologies (Invitrogen), Carlsbad, California, USA	Cat# 11913-019
PBS	Thermo Fisher Scientific, Waltham, Massachusetts, USA	Cat# 14190-169
HBSS	Thermo Fisher Scientific, Waltham, Massachusetts, USA	Cat# 14025092
**Plasmids**		
pcDNA™3.1 (+) Mammalian Expression Vector	Life technologies (Invitrogen), Carlsbad, California, USA	Cat# V79020
pcDNA3.1 FL-BAG6 and CT-BAG6	own lab	Pogge von Strandmann, Simhadri et al., 2007
pcDNA3.1 BAG6R786*	Own lab	This paper
pCS2-HRS	Addgene, Cambridge, Massachusetts, USA	Cat# 29685
pcDNA3.1-p53	kindly provided by Dr. Pattingre, INSERM, France	Sebti, Prébois et al., 2014
pcDNA 3.1-p53 K372-373R		
**Commercial kits**		
RNeasy mini Kit	Qiagen, Venlo, Netherlands	Cat# 74104
RNase-free DNase Set	Qiagen, Venlo, Netherlands	Cat# 79254
Pierce™ protein A magnetic beads	Thermo Fisher Scientific Waltham, Massachusetts, USA	Cat# 88845
Lexsy in vitro translation Kit	Jena Bioscience, Jena, Thüringen, Germany	Cat# EGE-2010-15
Matchmaker® Gold Yeast two hybrid system	BD Clontech, USA	Cat# 630489
ExoRNeasy Serum/Plasma Midi Kit	Qiagen, Venlo, Netherlands	Cat# 77044
Polystrene beads for EV flow cytometry	Polysciences, Inc, Warrington, Pennsylvania, USA	Cat# 17135-5
Zombie Green™ Fixable Viability Kit	Biolegend, San Diego, USA	Cat# 423111
Hs_TP53_7 FlexiTube siRNA	Qiagen, Venlo, Netherlands	Cat# SI02623747
Pierce™ BCA protein assay Kit	Thermo Fisher Scientific Waltham, Massachusetts, USA	Cat# 23225
KPL Histomark® Biotin Streptavin-HRP Systems	SerCare, Milford, Massachusetts, USA	Cat# 71-00-38
AEC (red) Substrate Kit	Life technologies (Invitrogen),	Cat# 002007

	Carlsbad, California, USA	
QuickChange II Site-Directed Mutagenesis Kit	Agilent Technology, Santa Clara, California, USA	Cat# 200524
Jetprime	peqLab, Polyplus transfections	Cat# 13-114-07
Lipofectamine® 2000 Transfection Reagent	Thermo Fisher Scientific Waltham, Massachusetts, USA	Cat# 11668027
RevertAid First Strand cDNA Sysnthesis Kit	Thermo Fisher Scientific Waltham, Massachusetts, USA	Cat# K1622
SYBR® Green JumpStart™ Taq ReadyMix™	Sigma, Kawasaki, Japan	Cat# S4438-100RXN
Experion RNA StdSens Analysis Kit	BioRad, Hercules, California, USA	Cat# 7007104
TruSeq Stranded mRNA LT kit	Illumina, San Diego, California, USA	Cat# RS-122-2101, RS-122-2102
Agilent High Sensitivity DNA Kit	Agilent, Santa Clara, California, USA	Cat# 5067-4626
**Experimental models: Cell lines**		
HEK293<br>HEK293 BAG6KO<br>HEK293 CBP/p300KO	DSMZ, Braunschweig, Germany<br>This paper<br>Sauer, Schuldner et al., 2017	Cat# ACC 635
B-16V<br><br>B-16V BAG6KO clones	DSMZ, Braunschweig, Niedersachsen, Germany<br>This paper	Cat# ACC 370
HCT116 and HCT116 p53KO	Annette Paschen, University Hospital Essen, Germany	
**Experimental models: Organisms/Strains**		
C57BL/6J	Charles River, Wilmington, Massachusetts, USA	
Eµ:Tcl1a;<br>Cd19:Cre;<br>Tp53fl/fl	Rickert, Rajewsky et al. 1995<br>Jonkers, Meuwissen et al. 2001<br>Knittel, Rehkmper et al., 2017	
**Deposited Data**		
Melanoma BAG6 expression data & survival data	The Cancer Genome Atlas, TCGA, National Cancer Institute, National Human Genome Research Institute	https://www.proteinatlas.org/ENSG00000204463-BAG6/pathology/tissue
**Microscopes and instruments**		
Immunohistochemistry microscope	Leica Typ DM1000 LED, Leica Microsystems, Wetzlar, Germany	

Immunefluorescence microscope	Leica Dmi8, Leica Microsystems, Wetzlar, Germany	
Flow cytometer	Gallios (Beckman Coulter, Brea, California, USA)<br>FACSCalibur (Becton Dickinson, Franklin Lakes, New Jersey, USA)	
real-time PCR system	7500 real-time PCR system, Life Technologies, Carlsbad, California, USA	
CCD camera	Intas ChemoCam Imager ECL, Type HR 16-3200, Intas Science Imaging Instruments GmbH, Göttingen, Niedersachsen, Germany	
Nanodrop	Nanodrop1000, Thermo Fisher Scientific, Waltham, Massachusetts, USA	
Nanoparticle tracking analysis	NS300, Malvern Instruments, Malvern, UK	
**Software and Algorithms**		
Graphpad Prism 6.0 software	GraphPad Software, Inc.	http://www.graphpad.com/scientific-software/prism/
FlowJo V10	FlowJo LLC<br>Treestar, Ashland, OR, USA	www.flowjo.com
RNAseq	STAR version 2.4.1a	
Functional annotation analysis	DAVID Bioinformatics Resources 6.8	https://david.ncifcrf.gov/
Protein interaction analysis	Search tool for the Retrieval of Interacting Genes/Proteins (String) database v9.0	http://www.string-db.org/
Immunoblot imaging software	Chemostar, Intas, Ahmedabad, Indien	
NTA analysis software	NanoSight NS300 Software NTA 3.1 Build 3.1.46, Malvern Panalytical, Malvern, UK	
Immunohistochemistry and fluorescence microscopy software	Leica Application Suite X (LAS X) and Imaris 8.3.1 software	
CRISPR sgRNA design Zhang lab	Cong, Ran et al. 2013, Ran, Hsu et al. 2013	http://crispr.mit.edu/
Mass spectrometry raw data	Maxquant, version 1.5.3.8	

analysis		
RNAseq library sequencing	digital PCR, QuantStudio 3D, Thermo Fisher Scientific<br>HiSeq 1500 platform, Illumina	
Raw transcriptome read alignment	STAR version 2.4.1a	
Heat map generation	web-enabled heat mapping for all. Nucleic Acids Res. 2016 May 17 (epub ahead of print). doi:10.1093/nar/gkw419	Babicki, Arndt, et al., 2016

Table S5. List of oligonucleotides

Oligonucleotide	Sequence 5'-3'
**sgRNA for CRISPR**	
sgRNA sequences targeting murine BAG6	GCTTGTAGGACCCGGCCC and GGGCCGGGTCCTACAAGC
**Primer for qRT-PCR**	
actin-for	ACACTGTGCCCATCTACGAGG
actin-rev	AGGGGCCGGACTCGTCATACT
Murine NFKBia-for	CTCAACTTCCAGAACAACCTGCA
Murine NFKBia –rev	GGAGCTCAGGATCACAGCCA
Murine Eno2-for	ACTCCGACCTCATCCTGC
Murine Eno2-rev	CTCTGCCCCAAGTCGCATG
Murine Murine HDAC7-for	CCTGAAGTTGCGCTACAAACC
Murine Murine HDAC7-rev	GAGGAATCTCCAAGGGTCTC
Murine Rgs16-for	TCAGTGAGGAGAACCTGGAGT
Murine Rgs16-rev	TCACCTCTTTAGGGGCTTCG
Murine Efemp2-for	CGAGCCTGATGAACAGGAGA
Murine Efemp2-rev	CGACACTCATCTATGTCCACAC
Murine Nes-for	AACAGAGATTGGAAGGCCGC T
Murine Nes-rev	AGGGACATCTTGAGGTGTGC
Murine Ptpn3-for	AGATGCCGCTCGTGGTCT
Murine Ptpn3-rev	AGGGCCAGTTCTCTCGAGT
Murine s100a9-for	TCGGCTTTGACAGAGTGCAA
Murine s100a9-rev	GCCCCAGCTTCACAGAGTAT
Murine Elane-for	CAGCAGGACCCACTGAGAAG
Murine Elane-rev	TTGTGCCAGATGCTGGAGAG
Murine MPO-for	CTGGTTAGCAGAGCTGGACC
Murine MPO-rev	GGGCCCATAAGTCAACCACA
Murine Prtn3-for	TTCTGCCGGCCACATAACAT
Murine Prtn3-rev	GCACATCCCCAGATCACGAA
Murine Mmp8-for	AAACTGTTCAGGACTACCTGG
Murine Mmp8-rev	ATTTGGCTTCCCCGTCACAT
Murine Ctnnal1-for	CAGAATGGCTGTGGCGAG
Murine Ctnnal1-rev	CACTCTCATCCGGTCAAAC
Human Ctnnal1-for	TCAGATGGAAAATAACGGATGGGT
Human Ctnnal1-rev	TGTTCCAGAGATCTGACCCCA

**Site-directed mutatgenesis primer**	
BAG6R786* for	CGGCTCCAGCCCCAGCTGTGATCCTTCTTCCACCAGCAC
BAG6R786* rev	GTGCTGGTGGAAGAAGGATCACAGCTGGGGCTGGAGCCG

1. Bichi R, Shinton SA, Martin ES, Koval A, Calin GA, Cesari R, et al. Human chronic lymphocytic leukemia modeled in mouse by targeted TCL1 expression. Proc Natl Acad Sci U S A. 2002;99(10):6955-60.
2. Rickert RC, Rajewsky K, Roes J. Impairment of T-cell-dependent B-cell responses and B-1 cell development in CD19-deficient mice. Nature. 1995;376(6538):352-5.
3. Jonkers J, Meuwissen R, van der Gulden H, Peterse H, van der Valk M, Berns A. Synergistic tumor suppressor activity of BRCA2 and p53 in a conditional mouse model for breast cancer. Nat Genet. 2001;29[6, 7] [6, 7] [6, 7]:418-25.
4. Knittel G, Rehkamper T, Korovkina D, Liedgens P, Fritz C, Torgovnick A, et al. Two mouse models reveal an actionable PARP1 dependence in aggressive chronic lymphocytic leukemia. Nat Commun. 2017;8(1):153.
5. Sauer M, Schuldner M, Hoffmann N, Cetintas A, Reiners KS, Shatnyeva O, et al. CBP/p300 acetyltransferases regulate the expression of NKG2D ligands on tumor cells. Oncogene. 2017;36(7):933-41.
6. Cong L, Ran FA, Cox D, Lin S, Barretto R, Habib N, et al. Multiplex genome engineering using CRISPR/Cas systems. Science. 2013;339(6121):819-23.
7. Ran FA, Hsu PD, Lin CY, Gootenberg JS, Konermann S, Trevino AE, et al. Double nicking by RNA-guided CRISPR Cas9 for enhanced genome editing specificity. Cell. 2013;154(6):1380-9.
8. Reinart N, Nguyen PH, Boucas J, Rosen N, Kvasnicka HM, Heukamp L, et al. Delayed development of chronic lymphocytic leukemia in the absence of macrophage migration inhibitory factor. Blood. 2013;121(5):812-21.
9. Pogge von Strandmann E, Simhadri VR, von Tresckow B, Sasse S, Reiners KS, Hansen HP, et al. Human leukocyte antigen-B-associated transcript 3 is released from tumor cells and engages the NKp30 receptor on natural killer cells. Immunity. 2007;27(6):965-74.
10. Reiners KS, Topolar D, Henke A, Simhadri VR, Kessler J, Sauer M, et al. Soluble ligands for NK cell receptors promote evasion of chronic lymphocytic leukemia cells from NK cell anti-tumor activity. Blood. 2013;121(18):3658-65.
11. Zerbino DR, Achuthan P, Akanni W, Amode MR, Barrell D, Bhai J, et al. Ensembl 2018. Nucleic Acids Res. 2018;46(D1):D754-D61.
12. Dobin A, Davis CA, Schlesinger F, Drenkow J, Zaleski C, Jha S, et al. STAR: ultrafast universal RNA-seq aligner. Bioinformatics. 2013;29(1):15-21.
13. Robinson MD, McCarthy DJ, Smyth GK. edgeR: a Bioconductor package for differential expression analysis of digital gene expression data. Bioinformatics. 2010;26(1):139-40.
